# Bioactive Glasses: Advancing Skin Tissue Repair through Multifunctional Mechanisms and Innovations

**DOI:** 10.34133/bmr.0134

**Published:** 2025-01-22

**Authors:** Zhiyang Ren, Shuhan Tang, Jia Wang, Shuqing Lv, Kai Zheng, Yong Xu, Ke Li

**Affiliations:** ^1^ Department of Burn and Plastic Surgery, The First Affiliated Hospital of Soochow University, Soochow University, Suzhou 215006, China.; ^2^ Suzhou Medical College of Soochow University, Soochow University, Suzhou 215123, China.; ^3^ Department of Orthopedic Surgery, The First Affiliated Hospital, Orthopedic Institute, MOE Key Laboratory of Geriatric Diseases and Immunology, Suzhou Medical College, Soochow University, Suzhou 215000, Jiangsu, China.; ^4^ Jiangsu Province Engineering Research Center of Stomatological Translational Medicine, Nanjing Medical University, Nanjing 210029, China.

## Abstract

As a complex and dynamically regulated process, wound healing is collaboratively carried out by multiple types of cells. However, the precise mechanisms by which these cells contribute to immune regulation are not yet fully understood. Although research on bone regeneration has been quite extensive, the application of bioactive glass (BG) in skin tissue repair remains still relatively underexplored. The review focuses on the principles and the latest progress of using BGs for skin tissue repair, highlighting BGs’ special performance requirements, including biological activity, biocompatibility, biodegradability, and antibacterial properties, emphasizing their potential for skin tissue repair. In addition, BGs play a substantial role in regulating various inflammatory cells (neutrophils, macrophages, mast cells, etc.) and tissue repair cells [fibroblasts, vascular endothelial cells, mesenchymal stem cells (MSCs), etc.] involved in wound healing. The review also covers recent developments in composite materials incorporating BGs, demonstrating their ability to promote angiogenesis, inhibit wound biofilms, and improve inflammatory responses in chronic wounds. Furthermore, BGs have shown effectiveness in promoting epithelial regeneration and collagen deposition in burn wounds as well as their applications in scar management and post-tumor resection wound care. Finally, we summarize our views on challenges and directions in the emerging field of BGs for skin tissue regeneration research in the future.

## Introduction

In daily surgical practice, particularly in trauma and reconstructive surgery, the repair of traumatic injuries, infectious defects, and deformities is a common and significant aspect of patient care. Human tissues can be damaged in terms of both morphology and function due to various factors, including mechanical trauma, chemical exposure, temperature extremes, and radiation [1,2]. In infectious disorders, diabetes mellitus (type II diabetes mellitus) can engender many complications as a comorbidity of the patient. Among the complications of diabetes, peripheral neuropathy is the most common, and about 50% of the patients will suffer from it all their lives [3]. These diseases cause skin and underlying soft tissue wounds, potentially leading to secondary chronic wounds. Chronic wounds encompass many human diseases with diverse etiologies, outcomes, treatments, and prognoses [4]. Timely intervention and therapy during the initial stages and during the late repair phase are critically significant in surgical practice. In these stages, deploying reagents, instruments, and dressings is essential for facilitating tissue regeneration.

Bioactive materials have come to the fore to address clinical necessities. As a rule, the definition of bioactive materials is a material that triggers specific biological reactions [5]. BG represents an important class of biomedical materials. Experimental and clinical studies have demonstrated that BGs have good biocompatibility, high biological activity, biodegradability, and bone repair properties [6], and garnered extensive broad focus within tissue regeneration and repair. Following the pioneering discovery of BG by Hench in 1971 [7], BG has been employed in bone or teeth tissue repair and regeneration [6,8] and has been identified important features as a bioactive system suitable for bone and dental repair and regeneration, such as high bioactivity, bone conductance, and bone stimulation. Given their inorganic nature and toughness, characteristics typically associated with “hard” tissues, it is not surprising that relatively little attention is paid to BG in soft tissue engineering. An early study by Wilson and colleagues [9,10] documented a stable interaction between BG and soft tissue regeneration, the earliest studies focusing on BGs in soft tissue repair. However, in recent years, scholars have shown that BGs can also induce up-regulation of genes related to wound repair, promoting tissue repair [8].

As a dynamic and highly regulated process, wound healing can be divided into 3 overlapping and interdependent stages: inflammation, proliferation, and remodeling. The inflammatory stage is a crucial phase for wound healing. Not only does it play a critical role in local immune response components, but also it has a decisive impact on the wound-healing process [11]. Importantly, moderate inflammation helps to remove necrotic tissue, kill local bacteria, and accelerate wound healing. However, excessively activated inflammatory responses can interfere with collagen deposition, angiogenesis, granulation, tissue formation, and other healing events [12]. The research results suggest that autophagy has a decisive impact that cannot be ignored on the acute exacerbation of chronic wounds during skin wound healing [13]. Improved hydration and reduced inflammation at the wound site are essential for appropriate autophagy regulation. Keratinocyte autophagy activates keratinocytes and fibroblasts and facilitates wound healing [14]. Therefore, this review has also focused on the regulatory effects of materials on cells involved in inflammatory response and subsequent immune regulation in the subsequent chapters.

We have reviewed recent articles on BGs and found that the application of this material in different diseases of skin tissue repair and its impact on related cells have not been systematically explained [15,16]. In this review, we have noticed the regulatory effect of BG on wound healing-related cells and further analyzed its specific mechanism of promoting healing. Additionally, we focused on the clinical application of BGs in acute and chronic wounds, burns, pressure ulcers, diabetes ulcers, scars, and even tumor suppression. At the same time, we systematically compared the composition materials and biological activities of different BGs and the toxicity of BG with different ions added and summarized the ideal BG material that releases ions without activity on human tissues. By providing this comprehensive overview, we aim to address a gap in the current literature on BG.

## Classification and Properties of BGs

### Classification

BGs are classified according to the primary glass formation in the glass network: silicate BG, borate BG, and phosphate BG [17,18] (Table 1). The BG with 45S5 composition (Table 1), commonly referred to as Bioglass, has been extensively studied for biomedical applications [18]. It is produced through a high-temperature melting process, which ensures its unique properties and effectiveness in medical use [19]. The range of BG compositions was further expanded based on the composition of 45S5 [20]. With ongoing advancements in preparation technology and evolving requirements, BG production has expanded to include various methods, such as the sol–gel process, hydrothermal synthesis, and template-based techniques [18,21]. The most representative of sol–gel-derived BGs are those based on ternary 58S and 77S composition systems (Table 1) [7]. Borate BG is primarily used in bone tissue repair [22] and has also demonstrated potential as a drug release matrix for treating bone infections [23,24]. However, research on using this type of BG as a wound-healing material has only been conducted in recent years [25].

**Table 1.** Chemical compositions (wt %) of extensively investigated BGs for biomedical applications

Forms	SiO_2_	P_2_O_5_	CaO	Na_2_O	MgO	K_2_O	CaF_2_	B_2_O_3_
45S5	45.0	6.0	24.5	24.5	0	0	0	0
58S	58.2	9.2	32.6	0	0	0	0	0
68S	67.5	9.1	23.4	0	0	0	0	0
77S	73.7	9.8	16.5	0	0	0	0	0
86S	85.7	9.0	5.3	0	0	0	0	0
13-93	53.0	4.0	20.0	6.0	5.0	12.0	0	0
A/W	34.0	16.2	44.7	0	4.6	0	0.5	0
13-93B3	0	3.7	18.5	5.5	4.6	11.1	0	56.6
S53P4	53.0	4.0	20.0	23.0	0	0	0	0
S70C30	71.4	0	28.6	0	0	0	0	0
P50C35N15	0	71.0	19.7	9.3	0	0	0	0

### Properties

BGs have good biological activity and biocompatibility. Specifically, the surface of BGs gradually develops a layer of bone-like nanocrystals known as hydroxyapatite (HA). This layer forms a binding interface with the host tissue, facilitating effective integration and bonding [26]. Their biological activity, degradability, and mechanical properties can be adjusted by changing the content of each component or adding other bioactive molecules to meet different clinical needs. In 1973, Hench and Paschall [27] reported that BGs could also be chemically bonded with soft tissue, and the subsequent research on bioactive repair of soft tissue has been carried out successively. Since most skin wounds are exposed or potentially infected, BGs have special performance requirements, such as biocompatibility, biodegradability, antibacterial properties, cell proliferation, and angiogenesis promotion.

#### Physicochemical properties

As mentioned earlier, a layer of HA nanocrystals will form on the surface of BGs. First, after immersing 45S5 in simulated body fluid (SBF) for 7 d, the material will gradually degrade, releasing ions such as Na^+^ and Ca^2+^ and converting to hydroxyl-carbonate-apatite (HCA) materials. The precipitated Ca^2+^ (binding to PO_4_^3−^/OH^−^ in physiological solutions) and released alkaline ions result in an elevated local pH, which has been well demonstrated in bone tissue engineering [28–30]. Studies have shown that sol–gel BGs such as 58S exhibit ion dissolution properties in SBF, demonstrating their good degradation performance in a physiological environment [31]. Experiments have proved that the degradation of borate BGs is about 10 times faster than 45S5 [32], making it one of the best matching materials for material degradation and tissue growth speeds. The mechanism of borate degradation to HA is similar to that of 45S5. The difference is that BO_3_^3−^ is highly soluble in water and does not form a silicon layer on the surface like silicate salts do. Instead, it completely transforms into an ionic state [33]. Due to its good degradability, BGs have unique advantages in the treatment of skin lesions. They do not need to be changed as frequently as traditional dressing, eliminating concerns about wound cleaning. In summary, both physiochemical and biological properties were altered to some extent by component modulation. The glass structure can be adjusted by changing its composition to realize the controllable adjustment of degradation rate, HA transformation ability, and various properties. Some additional trace elements, such as Cu, Zn, Co, and Sr, can not only improve the processing performance of glass but also gradually release various inorganic ions into the microenvironment of the implanted site with the progress of degradation, which can promote cell adhesion, proliferation, and differentiation, accelerate angiogenesis, and exert antibacterial and anti-inflammatory effects, as well as other physiological functions.

As mentioned above, BGs increase the pH of the surrounding environment solution during degradation [28,29], and the high pH environment has antibacterial effects. Stimulus-responsive biomaterials are triggered by the wound microenvironment or external factors, which have significant advantages in precision drug delivery and release [34]. Usually, BGs release Ca^2+^, P^5+^, and Si^4+^ during degradation. Maeno et al. [35] found that lower concentrations of Ca^2+^ (2 to 4 mM) were suitable for osteoblast proliferation, differentiation, and mineralization through extracellular mechanisms. However, higher concentrations of Ca^2+^ (>10 mM) caused cytotoxicity. The result also provides the BG application in the epithelial cells of enlightenment. P^5+^ can stimulate the expression of matrix Gla protein (MGP), which may affect the proliferation and migration of endothelial cells [36]. It may have an impact on the regulation of angiogenesis in skin wounds. The authors hypothesized that it may regulate angiogenesis in skin wounds, which is worth further research. It has been demonstrated that BGs release Si^2+^ ions to accelerate angiogenesis and play a role in chronic painless wounds [37].In addition, strontium-HA (Sr-HA) can be formed in the BG material when Ca is replaced by Sr, which is the key to promoting bone cell proliferation [38]. Notably, incubation with 0.001 g and 0.01 g of glass particles can significantly inhibit the growth of subgingival bacteria, actinomycetes, and porphyromonas gingivalis. The antimicrobial activity was dependent on the concentration of Sr [39].

Ions added to the grid framework also play a role in BG composites. CeO_2_ can be the outer and cytoplasm membranes, inhibiting bacterial growth [40]. Zheng et al. [41] prepared silver-modified mesoporous BG (MBG) nanoparticles and demonstrated their ability to inhibit bacteria. Another nanocrystalline glass-ceramic (GC) and Ag-doped GC powders were prepared and used in the electrostatic spinning manufacturing of nanofibers containing chitosan (Ch)/polyethylene oxide (PEO)/Gel stent, showing good results in resistance to infection [42]. In cytological studies, Cu^2+^ was found to up-regulate the expression of vascular endothelial growth factor (VEGF), which positively affects angiogenesis and accelerates the contraction and closure of skin wounds [43,44]. Li et al. [45] Incorporated copper-containing monodisperse BG nanoparticle (BGN) into hydrogels and demonstrated its ability to enhance angiogenic capacity and promote wound healing in diabetic patients. The experiments confirmed that BGs had an excellent drug delivery function. This “ion-driven” antibacterial approach is of great significance to overcome the resistance induced by antibiotics.

#### Biological properties

Biocompatibility means that materials have good affinity with human tissues and are nontoxic, carcinogenic, and teratogenic to human beings. Day et al. cocultured 45S5 with fibroblasts and found that its secretion of basic fibroblast growth factor (bFGF) was increased both in vivo and in the cutaneous wound model, which promoted cell proliferation, thus demonstrating its good biocompatibility [46]. Varmette et al. [47] studied the cytologic properties of sol–gel BG (58S), found its regulatory effect on macrophages, and demonstrated that its culture medium promoted cell proliferation, indicating good cytological activity for 58S. It is important to note that borate bioglass may potentially be biotoxic. As reported by Brown et al. [48], several kinds of BGs can trigger cytotoxicity under static culture conditions in vitro, but they are harmless to cells under dynamic conditions. This composition did not show any toxicity in vivo and instead promoted tissue regeneration in the rat model. In addition, Zhao et al. [49] added Cu^2+^ to the borate BG composition to promote vascularization, which is quite effective in enhancing the bioactivity of BGs. Therefore, the combination of BG materials has different effects on cell biocompatibility and cell viability.

It was concluded that ions, typical of borate glass release, are not toxic in dynamic environments such as the human body. These BGs with good cellular activity and degradability have great prospects in the related applications of soft tissue engineering, not only in the human body but also in other mammals, which can be used as one of the ideal materials for treating wound injuries. As mentioned in the study, BGs promote cytokine secretion, angiogenesis, and hemostasis and have good anti-inflammatory ability [15]. This is facilitated by the regulation of immune and inflammatory cells to promote wound repair and angiogenesis, which will be explained in the “Cell Regulation Involving BG” section.

### Formulations of BG-based wound dressings

Different from orthopedic injuries, skin wound dressings are mostly powder or paste. BGs used for skin damage are generally powdered and applied to the wound’s surface. Studies have shown that melt method 45S5 and sol–gel BG can promote wound healing, but sol–gel BGs can heal faster due to their particular structure [50]. Upon contact with the wound, BGs undergo a rapid ion reaction, forming a unique membrane-like structure. This structure not only functions to moisturize and promote hemostasis but also serves as an effective scaffold, facilitating the proliferation and adhesion of repair cells.

However, BG powder is easy to move and lose, making it difficult to fix at the tissue repair site [51]. Due to the irregular shape of the damaged tissue, it is necessary to fix the BGs with the polymer complex at the required site for tissue repair without affecting its biological activity. MBG (70S25C5P) fiber scaffolds with hollow fibers prepared by electrospinning can be used in skin tissue engineering. This glass fiber can not only be used as a scaffold for supporting tissue regeneration but also be loaded with antibiotics to play an antibacterial effect [52]. Cotton-wool-like BG fiber mats are a practical solution that uses a great variety of BG ingredients. These ingredients are usually prepared by sol–gel processes and hydrolysis of alkoxide precursors, which allows the formation of silicate glass networks (gels) from bottom to top at ambient temperatures [53], which is one way to solve the problem. Injectable agarose, alginate, and BG composite hydrogels were prepared based on the Ca^2+^ release during BG degradation, which can cross-link alginate molecules [54]. For wound-healing purposes, BG/human serum albumin (HSA) composite hydrogels were prepared by utilizing the high pH during BG degradation that activated the reaction of succinimide with amino groups. These bioactive ions released from BG/HSA composite hydrogel induce neovascularization and promote the healing of chronic wounds [55]. The hydrogel exhibits suitable tissue viscosity (approximately 81 kPa), providing strong adhesion to the skin even in aqueous solution while reducing the intensity of bacteria invasion. Researchers also prepared beneficial formulations using ointment and BG powder for the moist environment provided by the ointment, which facilitates wound process. In addition, to produce sturdy BG-based scaffolds with specific dimensions and high porosity, BG powder can be used for direct 3-dimensional (3D) printing for bone regeneration [16] (Fig. S1). Strategies regarding the use of these BGs have been reviewed previously.

## Cell Regulation Involving BG

**Fig. 1.**
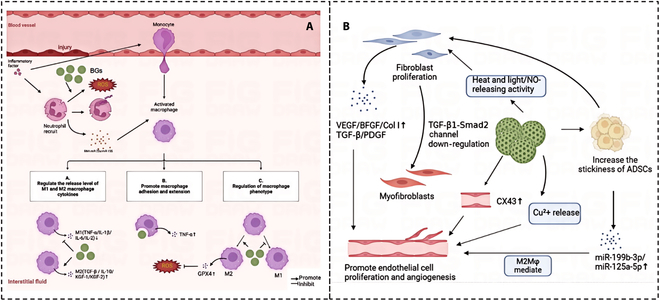
(A) BG acts on neutrophils, increasing free radicals and promoting wound activation. BG can be phagocytic by macrophages, promote adhesion and extension of macrophages, and up-regulate TNF-α. BG acts on macrophages to regulate their cytokine secretion, and when macrophages are activated as M1 macrophages, TNF-α, IL-6, and IL-10 are down-regulated. BG can regulate the phenotype of macrophages, inhibit the inflammatory response, reduce the activity expression of M1 macrophages, reduce inflammation, activate the transformation of macrophages to M2, express more anti-inflammatory factors, promote angiogenesis, up-regulate GPX4 expression, clear excess ROS, and improve mitochondrial function. (B) BG induces the expression of TGF-β and PDGF in fibroblasts, and the high expression of bFGF, VEGF, and Col I can promote endothelial cell proliferation and accelerate the process of angiogenesis, which regulates the activity of light and heat and nitric oxide release, promoting the proliferation of fibroblasts; BG also down-regulates collagen synthesis and fibroblast differentiation into myofibroblasts via the TGF-β1–Smad2 signaling pathway. BG can promote endothelial cell gap junction, promote CX43 expression, and accelerate the process of neovascularization. ADSCs self-renew and differentiate into keratinocytes, DFs, and cells in the basal layer, and they show strong migration and recruitment ability to the injured site. BG can enhance the adhesion of ADSC stem cells. BG pretreatment enhanced the therapeutic ability of mesenchymal stem cell-derived extracellular vesicle (MSC-EV) and significantly up-regulated functional substances such as miR-199b-3p and miR-125a-5p.

The wound-healing process is complex yet highly ordered, typically divided into 3 stages: inflammatory reaction, proliferation, and matrix remodeling [56]. Within these stages, various cells play crucial roles in wound healing. The cells involved in wound repair include various immune cells (neutrophils, macrophages, mast cells, etc.) [11] (Fig. 1A) and tissue repair cells (fibroblasts, vascular endothelial cells, MSCs, etc.) [57,58] (Fig. 1B). Therefore, the regulation of these participating cells by BGs will be explored in this section.

### BGs regulate neutrophils

Neutrophils are the primary cellular responders that gather in injured tissues and exert immune functions. They can not only kill pathogenic microorganisms but also accelerate the healing process of wounds [59]. After activation, neutrophils form prominent extracellular structures known as neutrophil extracellular traps (NETs) [60]. NETs will extend into the extracellular microenvironment to capture and kill pathogens by budding from the nuclear or releasing from the chromatin. However, neutrophils are essential for initial immune responses but can also hinder the healing process. They release soluble mediators and excessive reactive oxygen species (ROS), which may impede healing [61]. Additionally, neutrophils secrete particles containing pro-inflammatory microRNAs, such as miR-23a and miR-155, which can contribute to tissue damage. Therefore, it is crucial to clear neutrophils from the inflammatory microenvironment to improve the healing effect of the wound [11,62].

Maitz et al. [63] have demonstrated that BGs can stimulate the production of free radicals by neutrophils, which promotes wound activation [64]. However, this effect is a double-edged sword for wound healing. Additionally, it has been elucidated that the plasma protein albumin α2-HS glycoprotein (AHSG) inhibits HA-induced neutrophil stimulation and partially restores inhibitory activity to HA-adsorbed serum [65]. This also explains the regulation of neutrophils by BGs via plasma.

### BGs regulate macrophages

Following neutrophils, circulating monocytes rapidly migrate into tissues in response to signals generated by skin injury. Moreover, exposure to the local inflammatory microenvironment leads to the differentiation of monocytes into macrophages [56]. After tissue damage, macrophages clear necrotic tissue and pathogens through phagocytosis and release of inflammatory factors [58,66]. As they engulf numbers of apoptotic neutrophils, they will promote the transformation of the inflammatory microenvironment into a proliferative microenvironment [67]. When an immune response occurs, macrophages can differentiate into 2 types of cells on time, known as classically activated macrophages (M1 macrophages) and activated macrophages (M2 macrophages) [68]. M1 macrophages have secretory functions and can secrete tumor necrosis factor-α (TNF-α), interleukin-1β (IL-1β), IL-6, IL-12, and various other cellular inflammatory factors, which can clear antigens, fight against bacteria, promote inflammation, and inhibit the proliferation of inflammatory cells [69]. In the early stages of trauma, they play a crucial role.

Contrary to M1 macrophages, M2 macrophages have the function of secreting anti-inflammatory factors such as IL-10, transforming growth factor-β (TGF-β), KGF-1, and KGF-2, which promote cell proliferation and tissue regeneration [70,71] (Fig. S2). The role of M2 macrophages is crucial for tissue repair in the late stage of trauma. Appropriate regulation and activation can even achieve scar-free healing. Under pathological conditions (such as diabetes). The balance between these 2 types is disrupted, resulting in prolonged inflammation at wound sites [67], which makes it difficult for the wound to heal.

Recently, researchers have found that BG also impacts the behavior and activation state of macrophages. Earlier, Bosetti et al. [72] found that BG could be phagocytosed by macrophages, stimulate the adhesion and extension of macrophages, and up-regulate the expression of TNF-α. However, Day and Boccaccini [73] also found that 45S5 regulated macrophage secretion of TNF-α, IL-6, and IL-10 cytokines. The secretion of these factors was down-regulated when macrophages were activated to M1 macrophages. Barrak et al. [74] also found that the s53p4BG lysate reversed the up-regulation of proinflammatory markers and inhibited M1-type macrophage polarization. These studies suggest that BG down-regulates the activity expression of M1 macrophages, reduces inflammation, and thus accelerates wound transformation.

BGs also demonstrated a positive effect on the regulation of M2 macrophages and promoted wound healing. Dong et al. [75] discovered that BG products can activate macrophages to transform into M2 type, expressing more anti-inflammatory factors, and promoting angiogenesis. Another study has also indicated that BGs containing Se^2+^ can enhance mitochondrial function and induce M2 polarization reprogramming in macrophages, which was characterized by the regulation of M1-related genes (iNOS, CD86, TNF-α, and IL-1β) and M2-related genes (CD206, CD163, Arg-1, and IL-10) [76] (Fig. S3). By up-regulating the expression of GPX4, this effect can be mediated, and GPX4 can clear excess ROS, thereby improving mitochondrial function. Chen et al. [77] found that IL-4 receptor-mediated M2 polarization of macrophages enhances angiogenesis and promotes effective skin wound healing. Si–Ca–Cu nanoglass can enhance the expression of anti-inflammatory factors IL-4/IL-10, promote wound repair, and play an antibacterial function [78]. Studies have shown that BGs can regulate macrophage phenotype to inhibit inflammatory response. However, to determine its specific regulatory function, researchers need to further study it.

Recently, researchers have focused on exploring the regulatory effects of BG complexes on macrophages. Zhang et al. [79] successfully prepared BGNs-Man/Ag for treating intracellular infections in macrophages. For the interaction between BGNs-Man/Ag and macrophages, mannose modification ensures the macrophage targeting ability of BGNs-Man/Ag, enhancing the expression of intracellular ROS and mediating the M1 polarization of macrophages. Among them, mannose promotes macrophage activation, while BGN exhibits improved drug delivery capability and antibacterial efficacy. Notably, most studies have concentrated on regulating BG’s effects on macrophages in bone or dental pulp tissue, with relatively limited research on its role in wound repair.

### BGs regulate fibroblasts

As the main cells that make up the dermis, fibroblasts have a critical impact on multiple stages of wound repair. Fibroblasts emit relevant signals that mediate the closure/filling of other key wound cell types and defect/injury sites [67]. The process of early recruitment of inflammatory cells and platelets to the wound site and the activity of these cells at the beginning of wound development activate and recruit fibroblasts to the injured area around the 5th to 7th day [80]. Mechanical tension, TGF-β, and other cytokines can induce the differentiation process of fibroblast subpopulations migrating to the wound site into myofibroblasts [81]. Insufficient apoptosis or excessive differentiation of myofibroblasts in the late stages of wound healing can lead to wound contracture after injury [58,81,82]. Fibroblasts promote wound regeneration and repair by secreting bFGF, TGF-β, platelet-derived growth factor (PDGF), and various other growth factors [83]. Studies have revealed an indispensable immunomodulatory role of fibroblast-secreted exosomes in directing the dynamics of macrophage activation [84]. Exosomes enhance the sensitivity of macrophages to M1 and M2 polarizing stimuli while also accelerating the timely switch from M1 to M2 phenotype.

**Fig. 2.**
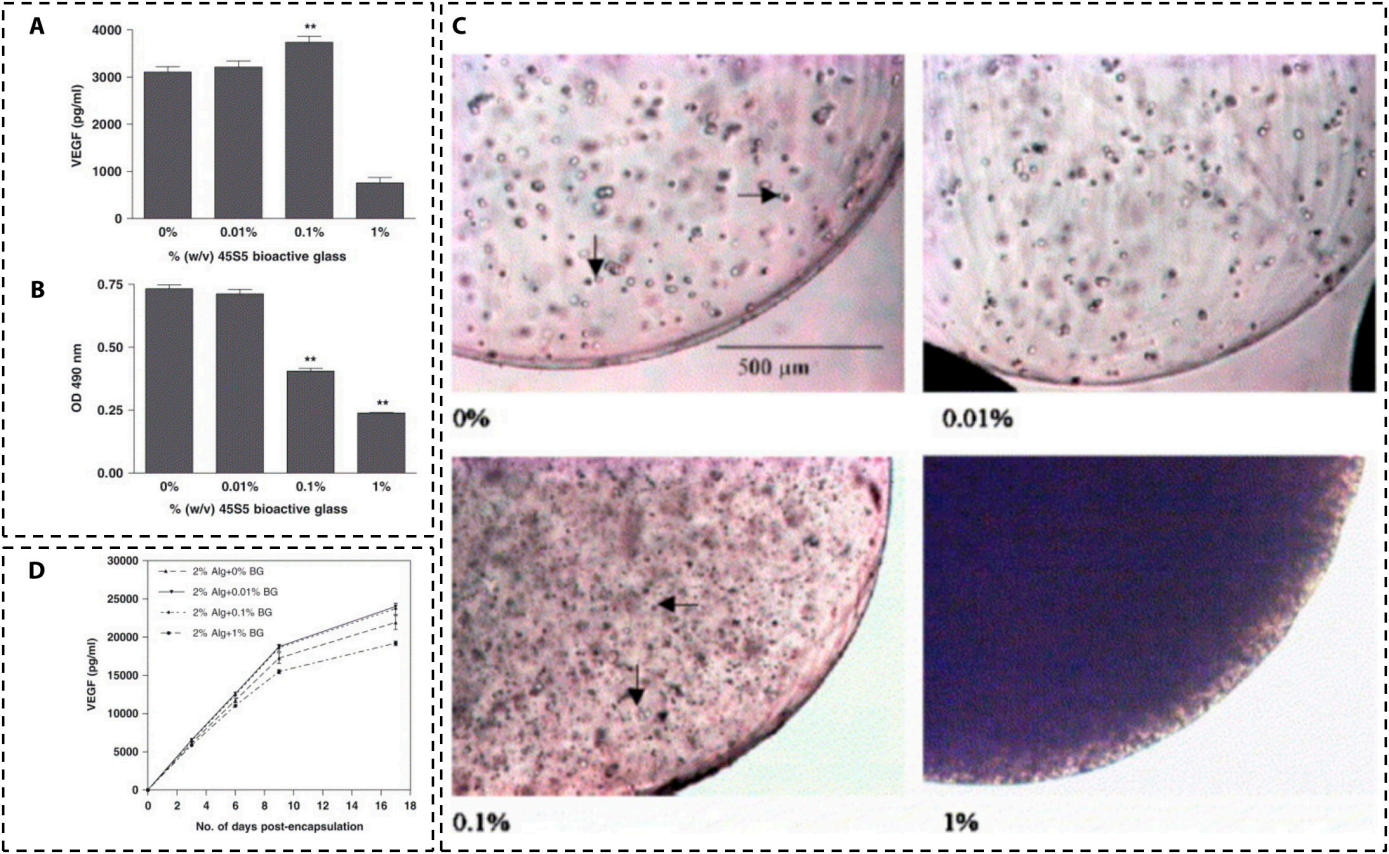
(A) VEGF secretion by CCD-18Co fibroblasts cultured on different amounts of 45S5 BG coating for 3 d. (B) Decreased number of metabolically active fibroblasts in cells cultured on surfaces coated with 45S5 BG. (C) Cells and 45S5 BG particles were uniformly distributed in the alginate microspheres. High-density glass particles at 1% (w/v) cause the beads to become opaque. (D) The higher concentration of glass (1%) resulted in less VEGF secretion. Adapted from [85].

Researchers have discovered that BG can regulate the growth, proliferation, secretion, migration, and differentiation of fibroblasts. In 2004, Day et al. [46] found that low concentrations (0.01 to 0.2 wt %) of 45S5 BG coating could promote fibroblast proliferation when cocultured with fibroblasts for 24 hours. It has been reported that human fibroblasts can secrete large amounts of VEGF at 0.01 and 0.1 wt % when cocultured with alginate coated with 45S5 BG (0.01 to 0.1 wt %) [85] (Fig. 2). Zhang et al. [86] also found that multifunctional chitosan (CS)/alginate saline gel combined with BG nanocomposites regulated photothermal and nitric oxide release activities to promote fibroblast proliferation. However, at a concentration of 1 wt %, the microspheres failed to stimulate fibroblasts to secrete VEGF [85], indicating that the response of fibroblasts to BG was concentration dependent. Yu et al. [87] found that BG can induce fibroblasts to highly express bFGF, VEGF, type 1 collagen (Col I), and other proteins that promote tissue repair. In addition, 45S5BG could also accelerate endothelial cell proliferation by isolating the culture medium of 45S5BG and fibroblasts [88], and through in vivo animal experiments, this has been confirmed [87]. Wang et al. [89] studied the effect of a Cu-doped BG composite scaffold on fibroblasts and found that it had good biocompatibility and could increase the angiogenic-related factors of fibroblasts and enhance the angiogenic ability of the scaffold. Sharifi et al. [42] also found that a BG-ceramic scaffold coated with zinc can promote fibroblast proliferation, which is expected to be used as a skin substitute. The latest study prepared a ternary composite hydrogel based on sodium alginate, carboxymethyl cellulose, and copper-doped 58S BG [90]. The expression of TGF-β, Col I, and VEGF increased after treatment, thereby preventing fibrosis and promoting angiogenesis. Pathology also observed increased epidermal thickness, fibroblast number, and collagen deposition. In addition, Chen and colleagues [91] used time-lapse imaging to show that 90S BG particles induced the migration process of fibroblasts and significantly inhibited fibroblast differentiation into myofibroblasts. Further analysis of intracellular signaling pathways revealed that 90S BG particles down-regulate collagen synthesis and fibroblast differentiation into myofibroblasts via TGF-β1–Smad2 signaling. One multi-crosslinked hydrogel built with hyaluronic acid–tyramine, thiolated glycol CS, and copan-doped BG was also found to have a function that can monitor cell migration the migration, proliferation, and differentiation of fibroblasts in skin wounds [92].

In conclusion, BG can enhance the proliferation and migration of fibroblasts and regulate the expression of relevant cytokines to promote wound healing.

### BGs regulate vascular endothelial cell

During the wound repair process, vascular endothelial cells promote and regulate neovascularization. Under the regulation of cells, growth factors, and adhesive substances, blood vessels in the body will form new blood vessels in ways such as sprouting, and vascular endothelial cells near the wound will divide and proliferate and finally evolve into capillaries [58,93]. Therefore, vascular endothelial cells can perform proliferation, differentiation, migration, and angiogenesis, among other physiological functions, with an obvious impact on wound healing.

**Fig. 3.**
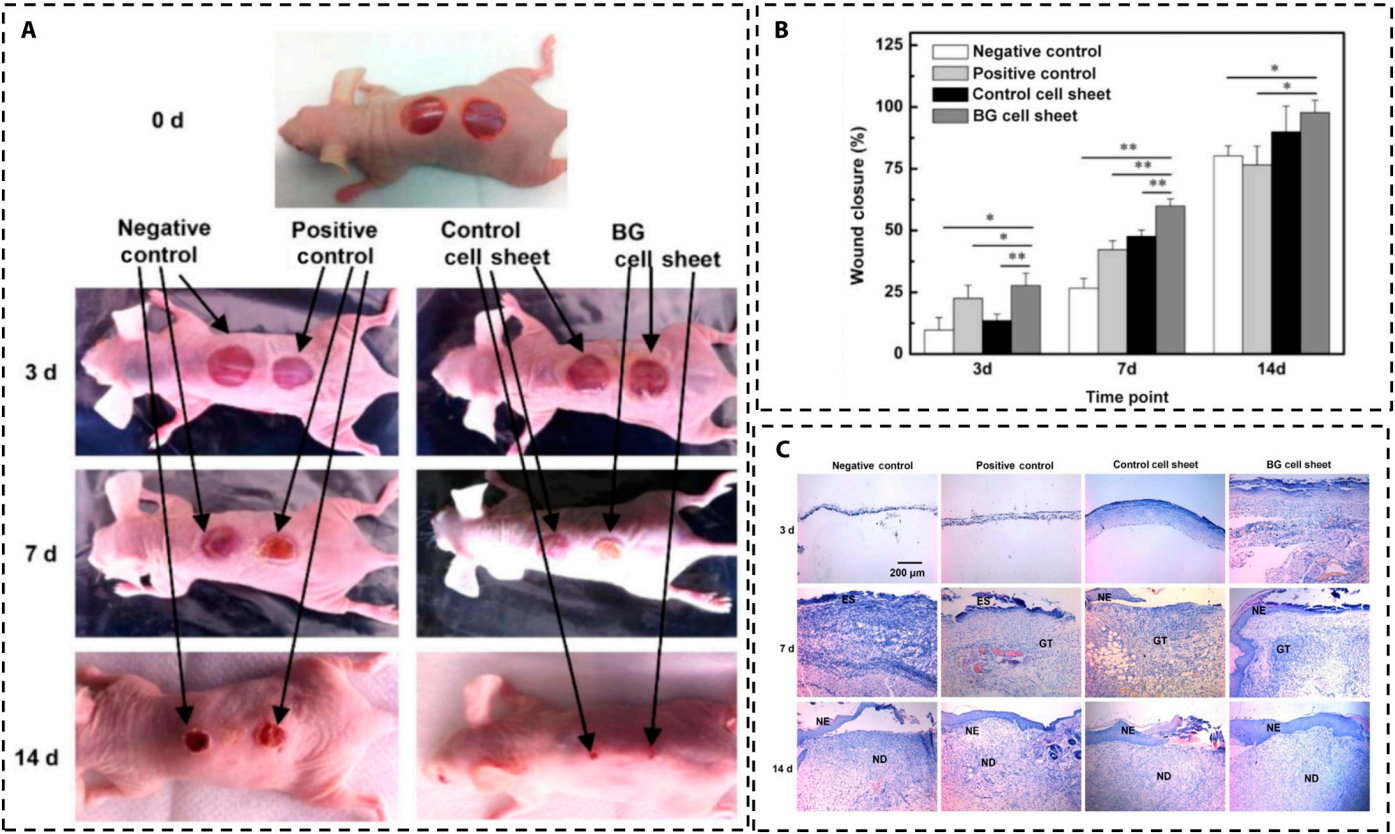
(A) Wound-healing condition on days 0, 3, 7, and 14. (B) Wound closure percentage in all groups, *n* = 3. (C) Images of hematoxylin and eosin (H&E) staining of sections on days 3, 7, and 14. The characteristics of wound-healing process with time are highlighted. ES, eschar; NE, neoepidermis; GT, granulation tissue; ND, neodermis. Adapted from [87].

Previous researchers have discovered that BGs can improve endothelial cell proliferation and angiogenesis [94]. Subsequent studies found that 45S5 could significantly increase the expression of fibroblasts and secrete more VEGF and bFGF, thereby activating human dermal microvascular endothelial cells, and it was found that their proliferation was significantly accelerated and the formation of vascular network was increased [46,87]. Yu et al. [87] also found that there were significant differences in the thickness of newly formed epidermis among all groups, and the thickest pathology showed the same results (Fig. 3).

Unfortunately, most studies on the regulation of BGs on vascular endothelial cells have focused on using BGs as a bone defect-related material to solve the problem of lack of angiogenesis in bone tissue [95,96], which reported that analysis of the response of VEGF-carrying BGs to tube formation by endothelial cells confirmed that immobilization of growth factors on BGNs using binding peptides can overcome the lack of neovascularization in the repair of large segmental bone defects [97]. Recently, the research on the effect of BG on endothelial cells in wound repair has been more and more in-depth. 45S5 BG was found to promote gap junctions and up-regulate the expression of CX43 in endothelial cells both in vitro and in vivo [98]. Hu et al. [25] developed a copper-containing borate BG dressing. It was found that after gradual degradation of poly lactic-co-glycolic acid (PLGA), some internal BG microfibers were exposed and reacted with body fluids to form a controlled and continuous Cu^2+^ release, which was observed in vivo with good vascular sprouting and remodeling. Zinc- and copper-doped mesoporous borate BGs can promote the migration and vascularization of endothelial cells [49,99]. One acid/silk fibroin dual-network hydrogel incorporated with BGNs suggested that it could have a positive impact on the migration of umbilical vascular endothelial cells in vitro [100]. When it was applied to full-thickness skin wounds, it was found that the collagen deposition was faster and arranged more neatly [49]. Li et al. [101] prepared BG/eggshell material and found that it could increase the levels of VEGF, its receptor 2 (KDR), hypoxia-inducible factor 1α (HIF-1α), and endothelial nitric oxide synthase (eNOS) and promote angiogenesis and induce wound repair in vivo. Solanki et al. [102] also designed composite fibers containing a novel BG to deliver cobalt ions at a stable rate, possibly due to the magnesium content of the glass, whose lysate stabilized HIF-1α and significantly increased VEGF expression, indicating that the composite activated the HIF pathway to stimulate vascular endothelial cells to promote angiogenesis.

In conclusion, BG can induce macrophages, fibroblasts, and endothelial cells in the wound to actively participate in wound repair, thereby promoting rapid wound repair. The specific mechanism needs to be further studied. It is worth noting that currently there are only a few reports on the mechanism by which BGs promote the barrier function of keratinocytes [103], as the cutin-forming cells in wound healing also remain a research problem to be solved.

### BGs regulate stem cells

MSCs have been widely confirmed to be a type of stem cells that remain in almost all adult organs, and they have the highest content in adipose tissue (AT). This kind of cells exhibit typical mesenchymal features and are isolated in the stromal vascular fraction (SVF) [104], which are mainly referred to as adipose-derived stem cells (ADSCs). Most reports have proved that ADSC can secrete rich secretome, thereby promoting cell proliferation and differentiation and migration and improving cellular and microenvironmental protection [105–107]. Recently, ADSC has been identified in subcutaneous tissues [108]. ADSCs self-renew and differentiate into keratinocytes, dermal fibroblasts (DFs), and other skin components in the basal layer [108,109]. These cells may affect the biological functions of damaged skin cells and exhibit a strong ability to migrate and be recruited to the site of injury [110,111]. Introducing ADSC based on stem cells as a crucial way to promote wound healing are widely used in preclinical applications [112], but even in the application of autologous or allogeneic environment, they also might not be able to reach the effect [113]. Therefore, it is feasible to study scaffolds that can be infiltrated by ADSC, using the mechanical and biochemical properties of biomaterials.

De Melo et al. [114] has customized a phosphate BG that can enhance stem cell adhesion, which is expected to become the new material to promote cell raise. In terms of noncellular transplantation, Xu et al. [115] found that BG pretreatment improved the healing efficacy of MSC-EVs and significantly up-regulated functional substances such as miR-199b-3p and miR-125a-5p, beneficial for regulating angiogenesis, mediated by M2 Mφ, in soft tissue injury model. So far, there are relatively few clinical trials combining BGs with MSC therapy in the field of skin injury, and we hope that more researches can be reported in this area.

## Application of BGs in Skin Tissue Repair

It is widely known that the largest organ in the human body is our skin. Epidermis plays a crucial role in repairing wounds resulting from trauma. When wounds become severe, prompt and effective treatment becomes essential. Currently, there is no ideal wound repair product that fully meets clinical needs. Specifically, ulcers caused by conditions such as pressure sores, varicose veins, vasculitis, and diabetes are often exacerbated by various factors, leading to chronic wounds. These refractory wounds significantly impact patients’ quality of life and can even pose serious health risks.

BGs have been shown to have a solid theoretical basis for promoting angiogenesis [46]. It stimulates fibroblasts to secrete various growth factors in vivo, enhances neovascularization, and supports endothelial cell proliferation [19,75]. Consequently, numerous wound dressings and ointments based on BGs have been developed to address clinical skin injuries.

### Application of BGs in acute trauma

As a widely researched key field, hemorrhage is closely related to battlefield injuries and life traumas. BGs can induce hemostasis by activating factor XII and other coagulation proteins [116]. Roy et al. [117] reported a unique BG composition, 70% SiO_2_; (30-*x*-*y*)% CaO; *x*: Al_2_O_3_; *y*: ZnO, where *x* = 10 to 18 mol % and *y* = 0 to 8 mol %, exhibiting hemostatic property as well as antibacterial activity. To evaluate cell toxicity, researchers conducted experiments and concluded that the prepared glass powder is nontoxic and harmless for the NIH3T3 cell line. In addition, in acute skin toxicity studies, it was observed that NIH3T3 cells remain in a high-energy state for a long time, suggesting excellent cell compatibility with Al-BAG. It shows that it has sufficient clinical application potential. At the moment of touching the blood, Al-BAG immediately triggers the coagulation cascade reaction in a “glass effect” manner, which is inherent to it, involving coagulation factor XII (Hageman factor), which stimulates and promotes the formation process of fibrin. It is likely that another coagulant is the polar silanol group on the surface of Al-BAG that contributes to the activation of factors XII and XI of the intrinsic pathway, stimulating and promoting fibrin production. Zinc is an essential cofactor in the processes of hemostasis and thrombosis [118]. For hemostasis and wound repair, Wang et al. [119] synthesized BGN with high CaO content using poly-tannic acid (PTA) and cationic antimicrobial polypeptide ε-polylysine (EPL) to functionalize BGN through layer-by-layer assembly. This significantly reduced the level of inflammation, ROS, and bacterial infection at the wound site, accelerated cell migration process, and stimulated angiogenesis, all of which can promote wound healing, which was more effective than using commercial BG (Dermlin) dressings.

In summary, the mechanism underlying the improved hemostatic effect of BGs remains unclear, and there have been no clinical studies on BGs in acute wounds both domestically and internationally. However, it is anticipated that the composition of BG will continue to serve as a highly effective hemostatic agent with antibacterial activity and can be utilized to treat both acute and chronic wounds [120].

### Application of BGs in chronic refractory wounds

Chronic refractory wounds are commonly known as ulcers. At present, there is no unified definition and description of chronic wounds in the world. Chronic refractory wound usually refers to a wound in a state of pathological inflammatory response that cannot achieve anatomical and functional integrity through a normal and orderly repair process under various internal and external factors [121]. The formation of chronic refractory wounds is mainly caused by insufficient angiogenesis, impaired nerve innervation, and cell migration disorders [120], including venous ulcer, ischemic ulcer, pressure ulcer, diabetic ulcer, infectious ulcer, radiation ulcer, and so on. How to promote chronic wound repair as soon as possible has become the difficult problem of surgical practice. Common measures mainly include debridement dressing, wound negative pressure drainage, and exogenous growth factors.

In the past 10 years, the application of BGs based on traditional surgical dressing has been proven to be very effective and basic and clinical studies have been carried out [122,123]. The most common preventable challenge to wound healing is infection. Biofilms consist of densely aggregated bacterial colonies coated by extracellular polymer (EPS) matrix, which in turn develop into chronic inflammation [124], making wound recovery difficult. Because antibiotics are difficult to diffuse within biofilms, Shirgill et al. [123] developed an AG-doped BG fiber that can inhibit biofilms. The glass fibers significantly reduced the biofilm viability (*P* = 2.08 × 10^−9^), which promoted the release of Ag directly from the biofilm.

A cobalt-containing BG fiber has also been found recently to shorten the healing time of chronic wounds [122]. The fiber morphology mimicked the morphology of extracellular matrix (ECM) fibrils. The expression level of HIF-1α clearly increased in the cobalt glass fiber group compared with the Dulbecco’s modified Eagle’s medium (DMEM) control group and DMEM containing the same amount of cobalt chloride. Keratinocyte cultures were exposed to fibrous conditioned medium, which showed that glass activated the HIF pathway and promoted VEGF expression.

The aqueous environment is essential for the release of bioactive ions from BGs [125]. Utilizing the combination of alginate and agarose may lead to the formation of a thermosensitive agar–alginate (AA) system with a high water content, with sufficient hydroxyl groups presenting in both polymers to provide sufficient water affinity to provide a humid environment [54]. BGs can maintain their biological activity in this high water content environment, better exert biological activities, regulate cellular performance and angiogenesis, and ultimately promote the healing of chronic wounds.

In the clinical cohort study, the patients were treated with Dermlin combined with nano-silver medical antibacterial dressing (nano-silver-MAD) [126]. Researchers found that the effective rate of the observation group was 95.12%, while that of the control group was 82.93%, which was significantly higher in the observation group than in the control group. Levels of VEGF and epidermal growth factor (EGF) were higher at 1 and 2 weeks after treatment in the observation group. It has been confirmed that the combination of Dermlin and nano-silver-MAD can significantly alleviate pain, accelerate recovery, and relieve inflammation. Therefore, BG has unique advantages and becomes a new direction for chronic refractory wounds.

#### Application of BGs in pressure ulcer wounds

Pressure ulcer wounds are localized injuries to the skin or subcutaneous tissue, often located at the bone carina, and usually caused by pressure or a combination of pressure and shear stress [127]. In 2020, a study was conducted and the results suggested that the global prevalence of pressure ulcers for the period 2008–2018 was 12.8%, ranging from 14.5% in Europe, 13.6% in North America, 12.7% in South America, 3% in Asia, 12.6% in the Middle East, and 9% in Australia [128]. One of the most widely recognized pressure ulcer classification systems is that of the National Pressure Ulcer Advisory Group (NPUAP). Stage 1 ulcers are only limited to the intact skin, but in stages 2 to 4, the wound gradually deepens, and the loss of skin and tissue also increases gradually; stage 2 pressure ulcers have skin shedding of partial thickness and exposure of the dermis; stage 3 often presents as full-thickness skin peeling and exposed AT can be seen; in stage 4 ulcers, full-layer skin and tissue loss often occurs with exposure of fascia, muscle, tendon, ligament, cartilage, or bone [129].

Studies have explored the effects of various surgical dressings on pressure ulcers from the perspective of evidence-based medicine [130–132]. This randomized controlled trial was designed to study adult patients with stage 2 or above pressure ulcers. From the experimental results, researchers found that the antibacterial function of the dressing is very significant, but there was a low difference in healing time between the dressing and medical gauze. Due to inflammatory infiltration, local skin ulceration and wound exudation were observed in stage 2 to 3 pressure ulcers [129]. Topical application of BGs can actively induce epithelial cell proliferation, effectively neutralize acidic products, and reduce exudation. Unfortunately, none of these experiments discussed the application of BG materials, and BGs have good properties in the hope of related reports in the future.

#### Application of BGs in diabetic ulcer wounds

As a metabolic disease, diabetes often causes chronic wound formation, which involves a series of complex pathophysiological mechanisms [133]. Nerve organic diseases, functional abnormalities, and vascular diseases of different degrees at the distal end of the lower limbs can cause a series of pathogen infections, skin ulcers, and deep tissue damage, leading to a common symptom, that is, diabetes foot. In the diabetic wound microenvironment, the wound of chronic diabetes is difficult to heal, and it has the main clinical characteristics of reduced angiogenesis, oxidative stress, bacterial infection, and others [134,135]. Foreign research data tell us that 40% to 60% of patients who need nontraumatic lower limb amputation are all due to diabetes, and 85% of diabetes-related lower distal amputations occur after foot ulcers [136]. Therefore, it is very important to accelerate the wound healing of diabetes foot ulcer and promote wound repair in clinic.

BGs have excellent biological activity, according to observation, whose product ion can stimulate cell migration and vascularization-related gene expression to promote healing of chronic wounds [11,75,92]. Studies have shown that BG can not only improve the barrier function of keratinocytes but also accelerate reepithelization, thus improving the wound healing in diabetes rats [103]. They found that BG extract significantly enhanced the barrier function of keratinocyte monolayers, with increased transepithelial electrical resistance and decreased paracellular permeability. In the in vivo model, the tight junctions of newly regenerated epidermis in the wounds of diabetes rats were increased. The previous article described that BG has a regulatory effect on macrophages. Research has shown that a dose-dependent modulation of macrophage proliferation/polarization and wound healing by BG particles was observed during full-thickness wound healing in diabetic rats [137]. Low concentration (20 μg ml^−1^) of BG particles can promote macrophage proliferation and induce the transformation from M1 to M2 phenotype. High concentrations (100 μg ml^−1^) of particles can prolong the existence time of M1 phenotype in macrophages while exhibiting significant cytotoxicity.

**Fig. 4.**
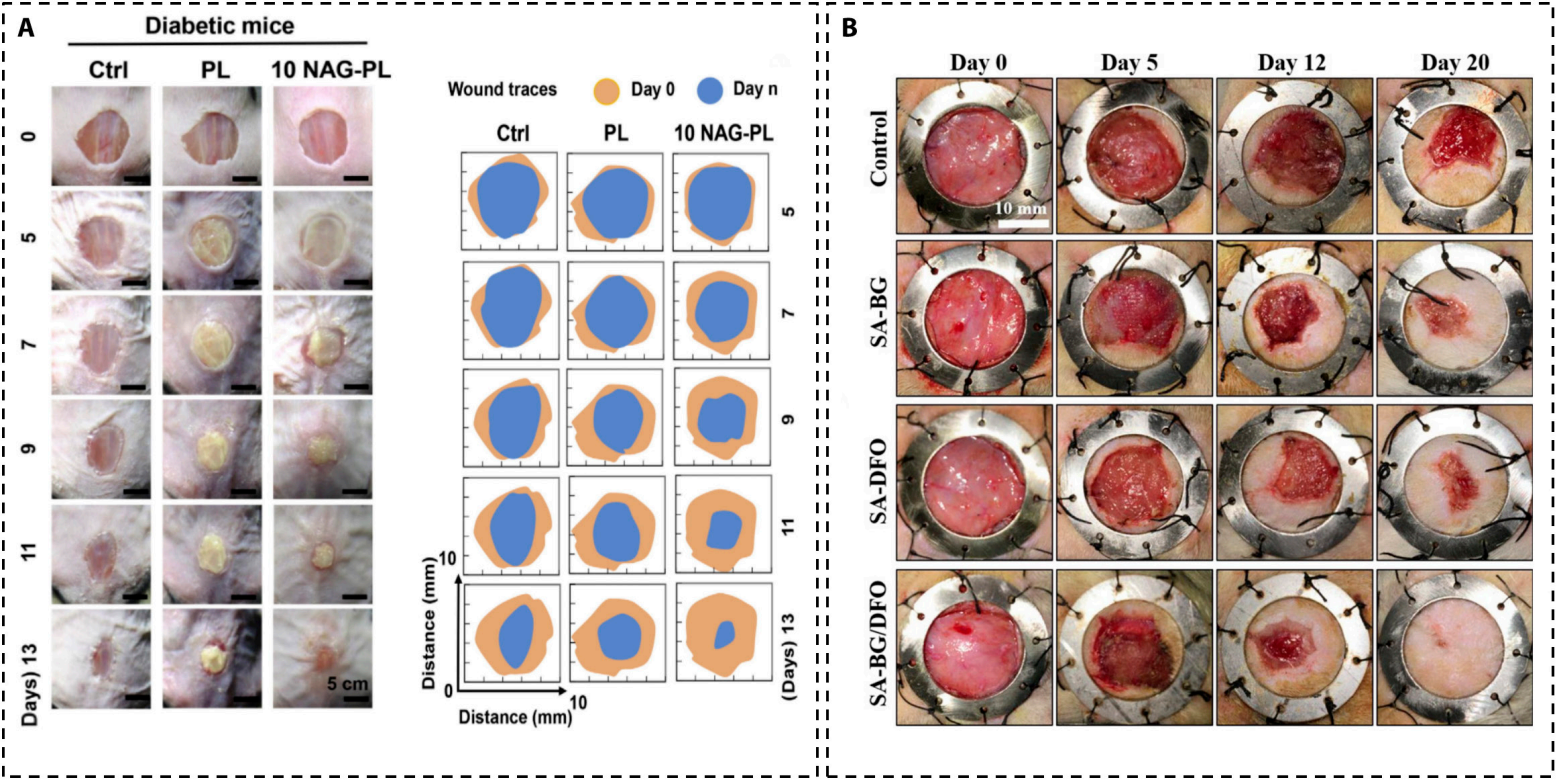
(A) Effect of the composite scaffolds containing NAG bioceramic particles on the diabetic wound healing. Adapted from [138]. (B) Bioactive injectable hydrogels containing desferrioxamine and bioglass for gross observation of wound healing. Adapted from [145].

With the study of BGs, BG ionic compounds were developed to solve the clinical demand. Nagelschmidtite (NAGEL, Ca_7_Si_2_P_2_O_16_), a BG containing Si, Ca, and P, was prepared by a domestic team through a co-electrospinning process [138]. In order to evaluate the effect of the composite stent on wound healing in vivo, the team conducted a wound-healing test for diabetes, treating wound areas of diabetic mice with PL, 10 nagelschmidtite-PL (NAG-PL) scaffolds, and control (undressed) (Fig. 4A). The cure rate of the treatment group (94%) was significantly higher than that of the control group (82%). The scaffold also significantly improved keratinocyte migration and ultimately accelerated and improved the formation of new epidermis in diabetes wounds, which solves the dilemma of ischemia and insufficient angiogenesis in diabetic wounds [139,140]. Yunnan BaiYao (a famous Chinese traditional Chinese medicine)/BG composite paste has also been pointed out to promote granulation tissue formation [141], reduce the inflammation of the wound, promote angiogenesis, and thus promote diabetes painless wound healing. Wu’s team [142] fabricated a nanocomposite dressing that combines a BG nanocoating with a pattern electrospun membrane (BG/PEM) by pulsed laser deposition (PLD) technology. BG/PEM can promote the expression of proliferation, adhesion, and angiogenesis-related genes such as eNOS and VEGF in human umbilical vein endothelial cells. In vivo research found that the wound-healing rate of diabetes mice treated with BG/PEM was close to 80% on the 13th day, obviously higher than that of the PEM group (57%) and control group (56%). In conclusion, because of its practical function in promoting angiogenesis and healing diabetes wounds, we believe that BG material can be regarded as a promising biomaterial.

However, due to their brittleness, BGs are challenging to use as substitute for skin and skin tissue engineering scaffolds [50]. Direct contact with BGs can potentially adhere to the wound bed, leading to issues such as tearing in diabetic wounds and other adverse effects [37].

Despite these challenges, novel composite dressings incorporating BG components show promise as effective biomaterials for promoting chronic wound healing. A bioactive layer consisting of silicate BG particles was placed on the modified Janus membrane to stimulate angiogenesis and wound healing [37]. After bioactive ions were released from the membrane, reflux was delivered to the wound bed through the modified Janus membrane. Gao et al. [143] also reported using bone ECM biomimetic cell-free nanofibrous scaffolds to promote full-thickness wound healing of diabetes. This bioactive nanofibrous matrix was made up of 3 types of substances, namely, ECM-componential collagen (Col, mimicking protein), polycaprolactone (PCL), and BGNs (mimicking biological apatite) (CPB). The last had the function of improving endothelial cell attachment and proliferation, increasing CD31 expression in the diabetic rat model. The mRNA and protein expressions of HIF-1α, VEGF, Col I, and α-smooth muscle actin (α-SMA) were significantly up-regulated, and angiogenesis was significantly enhanced. In addition, effective diabetic wound healing was observed in the CPB group due to rapid angiogenesis, granulation tissue formation, collagen matrix remodeling, and accelerated epidermal differentiation. A variety of synthetic biomaterials can be used to prepare hydrogels, including polyacrylic acid, polyacrylamide, and its derivatives, and natural biomaterials, including alginate, collagen, and CS [144]. With the in-depth research on polysaccharide materials, some hydrogel composite BG materials have been prepared to repair diabetic wounds. Desferrioxamine (DFO) can up-regulate angiogenic factors to promote revascularization (Fig. 4B). A DFO/ BG composite hydrogel system promotes angiogenesis and diabetic wound healing by promoting endothelial cell migration [145]. Li et al. [146] incorporated copper-containing monodisperse BGN into hydrogels and demonstrated that it could enhance angiogenic capacity and promote wound healing in diabetic mice. Shang et al. [147] prepared a chitosan nanoparticles, MSC-derived, BG, and TiO_2_ (CMCS-CEBT) hydrogel, known as a synthesis from exosome-encapsulated carboxymethyl chitosan (CMCS), CS nanoparticles (CS-NPs), BG, and TiO_2_ nanoparticle. In vitro analyses, it has been showed that the hydrogel had excellent cytocompatibility, stimulated endothelial cell adhesion and proliferation, and had anti-inflammatory, angiogenic, and antimicrobial activities. Composite hydrogel dressing can accelerate wound closure and repair in vivo, stimulate vascular regeneration, promote collagen deposition, and up-regulate the expression of anti-inflammatory factors. Similar results have also been achieved using CS-BG (Na-free) scaffolds in diabetic rats [148]. Chen et al. [149] prepared a multifunctional injectable hydrogel loaded with cerium-containing BGNs to promote wound healing in diabetes. This study makes good use of the 3D structure, swelling ratio, and compressibility of hydrogel, which can create a more beneficial environment and further improve wound healing. In addition, the sol–gel method combined with the template method is used to synthesize Ce-BG. CeO_2_ released from Ce-BG also exhibited deoxyribonuclease (DNase) mimetic activity [150]. It may utilize eDNA to perform cleavage functions and clear biofilms. In addition, it has been confirmed that H_2_O_2_ shows antibacterial effects at low concentrations and promotes angiogenesis in wound healing [151,152]. However, high concentrations of H_2_O_2_ may lead to prolonged healing time by inducing ROS to induce endothelial damage [153]. Therefore, in clinical practice, continuous monitoring and appropriate adjustment of H_2_O_2_ concentration are of great significance for wound repair in diabetes. Huang et al. [154] have already succeeded in preparing a multifunctional GelMA hydrogel incorporated with MnBG particles and CePO_4_:Tb nanomaterials as a H_2_O_2_-responsive smart hydrogel wound dressing, which could monitor the concentration of H_2_O_2_ in the wound microenvironment while clearing H_2_O_2_, thus accelerating the process of tissue repair and wound closure in diabetes. Unusually, Bargavi et al. [155] invented one multi-functional bandage (a BG/metal oxide/alginate composite-based regenerative membrane) and is verified by zebrafish and simulation of diabetic rat. Incorporation of Al_2_O_3_-TiO_2_ in nanostructured BG can control bacterial infections and promote tissue repair and wound closure.

All in all, BGs can improve the healing rate of diabetic foot ulcers and significantly shorten the healing time. At the same time, BGs can be used together with other new dressings to make the wound heal in a relatively wet environment, which is conducive to the dissolution of necrotic tissue and reduces infection. Therefore, BG composite scaffold is a kind of bioactive material with excellent development prospects that can be used for chronic wound-healing applications.

#### Application of BGs in burn ulcer wounds

**Fig. 5.**
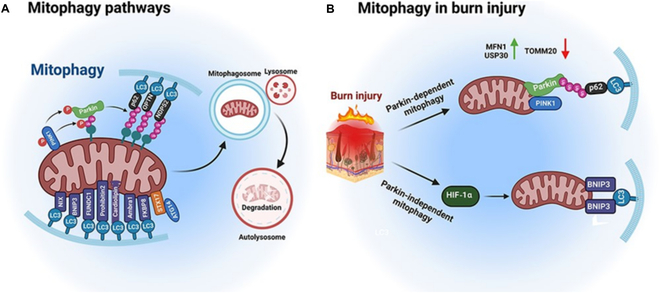
Overview of autophagy pathways in normal and burned tissues. (A) Mitochondrial autophagy is a significant mechanism by which cells regulate mitochondrial quality, including selective removal of damaged mitochondria through autophagy. The PTEN-induced kinase (PINK1)–Parkin pathway is the most extensively studied pathway for mitophagy. (B) Burns can not only trigger Parkin-dependent autophagy but also induce Parkin-independent autophagy. Activated Parkin interacts with Pink1 to initiate mitophagy. Moreover, Parkin-independent mitophagy can be induced by HIF-1α and BNIP3. Adapted from [165].

Burn ulcers refer to deep burn wounds that cannot heal quickly or that require debridement, anti-infection, skin grafting, and other treatment measures due to infection. In the United States, approximately 1.1 million burn patients are admitted to the hospital each year, of whom approximately 660,000 are diagnosed with second-degree burn injuries [156,157]. Second-degree burns are characterized by impaired integrity of the entire epidermis, varying dermal depth, and often require immediate medical attention [158]. Depending on the degree of injury, burns can cause various complications, including infection, hypothermia, scar formation, and bone and joint problems [159,160]. Among these difficulties, bacterial infection is the main cause of death after extensive burns, and patients with burns show a greater likelihood of multidrug resistance [161]. Second-degree burn wound treatment in the clinical practice of nursing standards includes thimerosal and top dressing, such as contact dressings and hydrogel dressing [162]. The primary goal is to promote optimal wound healing while relieving pain and preventing feelings. Specifically, BGs as topical therapy with good biocompatibility can be used to treat burns because plenty of local antibiotics commonly used for burns exhibit certain cytotoxicity, especially toward soft tissue cells [163], resulting in an unexpected delay in the wound-healing process. Kargozar et al. [164] also described the possibility of BG application to burn wounds. Notably, mitophagy is an important mechanism regulating the metabolic transition of macrophages in burn wounds [165] (Fig. 5). In gingival tissue, a molybdenum containing BGs has been shown to improve macrophage activity by regulating the function of mitochondria [166], which is an important theoretical basis for studying the clinical application of BGs in burn wounds.

From an antibacterial point of view, BGs can reduce the risk of infection of both Gram-positive and Gram-negative strains [39]. One research developed Ag-doped 70S30C glasses with 3D cotton-like structures and studied their antibacterial properties [167]. Kermani et al. [168] also synthesized a modified sol–gel of a mesoporous borate BG. The data showed that the undoped BGs had 50% antibacterial activity against both Gram-positive and Gram-negative bacteria. Incorporation of Ag into BGs resulted in a 98% increase in the antimicrobial activity of BGs against Gram-negative bacteria. Both of them are suitable for rescuing burn patients.

Zhu et al. [169] prepared a BG fiber loaded with platelet-rich plasma (PRP), evaluated it in a rat model of deep degree thermal wound, and reported that PRP^+^ nano-BG fiber promotes wound epithelialization mainly by promoting the expression of EGF, VEGF, TGF-β, HIF-1α, and integrin α3 and increasing the release of integrin β1 so as to accelerate wound-healing speed and improve wound-healing quality.

Hydrogel materials have received much attention in clinical practice in a variety of advanced dressings with soothing and moisturizing effects. Fayyazbakhsh et al. [158] fabricated a 3D-printed hydrogel with bioactive borate glass to slowly release water from the dressing and treat second-degree burn wounds. The treatment group had the highest hair follicle regeneration rate in the rat back second-degree burn model, indicating that bioactive borate glass (BBG) had a significant effect on hair follicle regeneration after burn. 3D-printed hydrogel-BBG20 dressing group implements the autolysis debridement, namely, noninvasive spontaneous removal of necrotic tissue, which also reflects the possibility of its application in burn wound repair. In order to solve the problem of insufficient adhesion in the cut part of the hydrogel [170], Paste is usually a mixture of various chemical substances. Its characteristics make it a rational choice for treating chronic and acute wounds and burns, also because of its good adhesion to the wound site [171]. Therefore, Li’s team [172] developed a paste, in which the chemicals used include CS, hyaluronic acid, 10 alginic acid, ZnO, and BG. Compared with BG or hydrogel materials, CS-BG materials exhibited higher biological adhesion and better antibacterial activity at suitable pH. More collagen deposition was also observed in an in vitro burn mouse model, which inhibited the expression of cytokines IL-6 and IL-1β, and regulated the levels of matrix metalloproteinases (MMPs), thereby exerting a certain influence on the healing process.

In clinical studies, Hu et al. [173] used BG and hyaluronic acid combined with bioactive material (combest) when treating burn wounds, and found that it has a favorable impact on the proliferation of granulation tissue and wound healing. The healing area of 18 patients in the trial group and 1 patient in the control group accounted for ^2^/_3_ of the wound area. The excellent and good rate of the experimental group was 95% (18 cases and 1 case), and that of the control group was 50% (1 case and 9 cases); the difference was statistically significant (*P* < 0.01). This study shows that burn wounds heal faster and repair is improved under the action of BGs, which is worth promoting widely in clinical practice.

In conclusion, the chemical composition of BGs is similar to that of natural elements in the human body, which is safe and stable. Due to its excellent antibacterial properties and various product forms, it has become a new direction for burn treatment.

To summarize this section, BGs can regulate cells involved in chronic wound repair, promote proliferation or secrete growth factors, and accelerate wound epithelialization or vascularization. Clinically, BGs can improve healing efficiency and shorten healing time. Table 2 provides a detailed summary of recent in vivo studies of BGs in chronic refractory wound disease.

**Table 2.** Summary of recent in vivo studies of BGs performed to different chronic refractory wound diseases

Chronic refractory wounds	Material	In vivo model/subject	Major outcome	Refs.
Diabetic ulcer wounds	58S-BG	Type I diabetic rat models	Accelerated re-epithelization; stimulated the keratinocyte differentiation	103
	Small particle size 58S-BG	Type I diabetic rat models	LBG promoted wound healing; HBG decelerated wound healing	137
	A conducive bioceramic (Ca7Si2P2O16)/PCB	Diabetic mice models	Improved epidermal regeneration, angiogenesis, and collagen deposition and decreased inflammatory response	138
	45S5 BG and Yunnan BaiYao ointments	Diabetic rat models	Accelerated the recovery	141
	BG/PEM nanocomposites	Diabetic mice models	Stimulated rapid angiogenesis and enhanced collagen deposition and re-epithelialization	142
	Bone ECM-biomimetic bioactive nanofibrous scaffolds (CPB)	Diabetic rat models	Improved blood vessel formation and epidermis differentiation	143
	Bioactive injectable hydrogels containing desferrioxamine and bioglass	Diabetic rat models	Promoted the expression of VEGF and HIF-1α and revascularization	145
	BGNC hydrogel scaffold composed of PEGDA and alginate	Diabetic mice models	Enhanced HIF-1α/VEGF expression and collagen matrix deposition	146
	CMCS-CEBT	Diabetic mice models	Enhanced vascularization and collagen deposition and exerted strong anti-inflammatory effects	147
	Chitosan-BG (Na-free) scaffolds	Type 2 diabetic rat models	Accelerated the recovery	148
	Ce-BG/GelMA hydrogels	Diabetic rat models	Enhanced wound-healing speed and reconstructed the skin tissue	135
	CPT-MnBG-Gel hydrogels	Diabetic rat models	Monitored the H_2_O_2_ concentration and accelerated wound healing	154
	45S5 BG/ Al_2_O_3_-TiO_2_ multi-functional bandage	Zebrafish models and type 2 diabetic rat models	Achieved anatomical fin regeneration, rapid re-epithelialization, and wound closure	155
Burn ulcer wounds	BG fibers loaded PRP	Deep second-degree thermal wound rat models	Promoted the wound epithelization through increasing the expression of EGF, VEGF, TGF-β, HIF-1α, integrin α3, integrin β1, and other mechanisms	169
	3D-printed hydrogels dressings with bioactive borate glass	Second-degree burn wound rat models	Continuous hydration, faster wound closure, and more homogeneous re-epithelialization	158
	CS-45S5 BG	Second-degree burn wound rat models	Promoted wound healing and reduced inflammation	172
	Combest combined with Bioglass and hyaluronan	Deep degrees II, granulated wounds, and graft site wounds patients	Benefited the proliferation of the granulation and wound healing	173

### Application of BGs in scar treatment

The pathological feature of hyperplastic scar is persistent inflammation, secondary ROS, and immature blood vessels and fibrous tissue hyperplasia [174]. Regulation of local mechanical stress is an effective way to manage postoperative scars, such as tape and pressure clothing that reduce tension [175]. In the animal model of hypertrophic scar, exhaustion of systemic macrophages effectively inhibited the formation of subacute hypertrophic scars during wound healing [176]. As mentioned above, BGs can effectively inhibit the M1 polarization of macrophages [74], which also demonstrates the potential and development prospects of BGs in scar-free wound healing.

Currently, there is a paucity of scholarly inquiries focusing on the research of BG in scar-free wound healing. Xiang et al. [177] prepared MBGs equipped with VR23 (a quinoline-sulfonyl hybrid proteasome inhibitor) by electrospinning technology, which inhibited scar formation by exerting the anti-inflammatory and anti-proliferation properties of the material. However, this study has certain limitations. The rat model used has a high self-healing ability, while collagen overdeposition usually occurs 4 to 6 weeks after surgery. The potential mechanism of BGs in inhibiting scar hyperplasia needs to be thoroughly explored in the future, which may include its anti-inflammatory effects, regulation of macrophages, regulation of fibroblasts, and influence on ECM.

### Application of BGs in surface tumor suppression

For certain types of surface tumors, surgical resection remains the primary treatment. Following surgery, patients often experience suboptimal physiological conditions, and changes in the tumor microenvironment, along with potential bacterial infections, can impede skin healing [178]. Therefore, materials that combine effective bacteriostatic and tumor-suppressive properties to improve tissue repair and wound closure are valuable to be applied to clinical practice.

Due to the good biological characteristics and spatial structure of BGs, a multitude of BG complexes has been used to address the aforementioned clinical issue. Due to the high drug loading efficiency of MBG, MBG loaded with 5-fluorouracil (5-FU) (an antitumor component) showed better antitumor ability than the MBG group and 5-FU group in vitro [179]. It was also confirmed that MBG-U core-shell nanofibers (CSF) could effectively inhibit tumor recurrence and promote wound healing by establishing an intact tumor resection mouse model. Chen et al. [180] also reported fetal bovine serum (FBS)-modified europium-doped BGNs (EuBGN@FBS), which can improve targeted therapy for tumor treatment and also assist in tumor imaging.

By the way, hydrogels can be well applied to wounds. Injectable hydrogel materials containing BG and other antitumor drugs have been developed. A multifunctional branched bioactive Si–Ca–P–Mo glass-ceramic nanoparticle (BBGN) with multifunctional branched bioactivity, in which molybdate nanocrystals are embedded, is reported for effective postoperative melanoma treatment or infection treatment and skin defect reconstruction [181]. Mo^4+^ and Mo^6+^ exhibit intentional photothermal properties under near-infrared (NIR) light stimulation and can combine good tumor photothermal therapy (PTT). Because of the good biocompatibility of BGs and the antitumor effect of Mo, BBGN-Mo could effectively inhibit tumor recurrence (96.4%) in the incomplete tumor resection model, improve anti-inflammatory and vascular microenvironment in the long term of the wound, and significantly promote postoperative skin regeneration. Huang et al. [182] and Liu et al. [183] also successfully developed injectable hydrogels for photo-thermochemical kinetics in synergistic tumor therapy. Mn^2+^ in BGs can activate the cGAS-STING immune pathway, resulting in a strong immune response. In addition, the system has good photothermal properties, promoting STING’s activation and reversing the tumor immunosuppressive microenvironment.

## Conclusions and Perspective

In summary, researchers in the field of tissue engineering have shown a strong interest in BGs and their derivatives. Experimental and clinical studies have shown that BGs have good biocompatibility, biodegradability, and bone-regenerating properties. However, their effect on soft tissue repair is less well understood and lacks comprehensive theoretical backing. For example, the acid-base imbalance in wounds caused by high concentrations of BGs during degradation may not always support wound healing [85]. Therefore, challenges in commercializing BG-based biomaterials for skin tissue repair remain.

Worth mentioning, it is still challenging to control the material composition, 3D structure, and mechanical strength precisely to meet the pathophysiological process of wound healing. Wound healing is a highly dynamic process involving various types of cells, cytokines, and ECM components. Therefore, researchers are increasingly focusing on how the tissue microenvironment regulates and guides the tissue regeneration process at different stages. Remarkably, the mechanical properties of biomaterials, such as stiffness and viscoelasticity, have a critical impact on accelerating tissue repair wound healing and warrant significant attention. However, most of the time, biomaterials composed of other synthetic polymers are responsible for regulating mechanical signals, and the mechanical properties of BG-based biomaterials have been largely overlooked. Given the unique physical and chemical properties of BGs, it is worthwhile to discuss the development of smart biomaterials that leverage BGs to control mechanical properties and adapt to the tissue microenvironment.

In terms of cellular regulation, in general, BGs can stimulate neutrophils, macrophages, fibroblasts, endothelial cells, and other related cells at the wound site. This stimulation enhances their participation in wound repair, thereby promoting the healing process. At present, there are relatively few reports on the regulatory effects and mechanisms of BGs on immune cells such as macrophages. Therefore, further research is needed to explore how BGs influence macrophage behavior, phenotypic changes, and the underlying mechanisms involved. In addition, BGs have the ability to promote the proliferation and migration of fibroblasts and endothelial cells, and can also enhance the expression of growth factors.

Furthermore, in the treatment of acute wounds and chronic refractory wounds, BGs have demonstrated significant potential in stimulating angiogenesis and promoting wound healing. In addition, the chemical composition of BGs closely resembles the natural inorganic elements found in the human body, ensuring safety and stability. BGs have excellent ion-loading capacity and exhibit multifunctional biological properties, such as antibacterial activity, which can enhance wound healing while reducing the risk of infection. These advantages make BGs a promising approach for chronic wound treatment. However, scientists need further experiments to thoroughly explore their structure and biological characteristics for biomaterials to be developed with clinical potential. Although most of these BG-based biomaterials are not yet ready for clinical applications, there is optimism that overcoming current challenges could facilitate their transition from the laboratory to clinical settings.

## References

[1] Tolles J. Emergency department management of patients with thermal burns. Emerg Med Pract. 2018;20(2):1–24. 29369586

[2] Zwierello W, Piorun K, Skorka-Majewicz M, Maruszewska A, Antoniewski J, Gutowska I. Burns: Classification, pathophysiology, and treatment: A review. Int J Mol Sci. 2023;24(4):3749. 36835171 10.3390/ijms24043749 PMC9959609

[3] Born LJ, Quiroga LH, Lagziel T, Hultman CS, Asif M. Clinical outcomes in ’diabese’ burn patients: A systematic review and meta-analysis. Burns. 2022;48(2):281–292. 34782233 10.1016/j.burns.2021.04.001

[4] Lim HW, Collins SAB, Resneck JS Jr, Bolognia JL, Hodge JA, Rohrer TA, Van Beek MJ, Margolis DJ, Sober AJ, Weinstock MA, et al. The burden of skin disease in the United States. J Am Acad Dermatol. 2017;76(5):958–972 e2. 28259441 10.1016/j.jaad.2016.12.043

[5] Reis RL. 2nd Consensus conference on definitions on biomaterials science. J Tissue Eng Regen Med. 2020;14(4):561–562. 32027470 10.1002/term.3016

[6] Hench LL, Polak JM. Third-generation biomedical materials. Science. 2002;295(5557):1014–1017. 11834817 10.1126/science.1067404

[7] Hench LL. The story of Bioglass®. J Mater Sci Mater Med. 2006;17(11):967–978. 17122907 10.1007/s10856-006-0432-z

[8] Miguez-Pacheco V, Hench LL, Boccaccini AR. Bioactive glasses beyond bone and teeth: Emerging applications in contact with soft tissues. Acta Biomater. 2015;13:1–15. 25462853 10.1016/j.actbio.2014.11.004

[9] Wilson J, Pigott GH, Schoen FJ, Hench LL. Toxicology and biocompatibility of bioglasses. J Biomed Mater Res. 1981;15(6):805–817. 7309763 10.1002/jbm.820150605

[10] Wang M, Hench LL, Bonfield W. Bioglass® high density polyethylene composite for soft tissue applications:: Preparation and evaluation. J Biomed Mater Res. 1998;42(4):577–586. 9827682 10.1002/(sici)1097-4636(19981215)42:4<577::aid-jbm14>3.0.co;2-2

[11] Wang Z, Qi F, Luo H, Xu G, Wang D. Inflammatory microenvironment of skin wounds. Front Immunol. 2022;13: Article 789274. 35300324 10.3389/fimmu.2022.789274 PMC8920979

[12] Huang C, Dong L, Zhao B, Lu Y, Huang S, Yuan Z, Luo G, Xu Y, Qian W. Anti-inflammatory hydrogel dressings and skin wound healing. Clin Transl Med. 2022;12(11): Article e1094. 36354147 10.1002/ctm2.1094 PMC9647861

[13] Mijaljica D, Spada F, Klionsky DJ, Harrison IP. Autophagy is the key to making chronic wounds acute in skin wound healing. Autophagy. 2023;19(9):2578–2584. 36994997 10.1080/15548627.2023.2194155 PMC10392758

[14] Qiang L, Yang S, Cui YH, He YY. Keratinocyte autophagy enables the activation of keratinocytes and fibroblasts and facilitates wound healing. Autophagy. 2021;17(9):2128–2143. 32866426 10.1080/15548627.2020.1816342 PMC8496719

[15] Mehrabi T, Mesgar AS, Mohammadi Z. Bioactive glasses: A promising therapeutic ion release strategy for enhancing wound healing. ACS Biomater Sci Eng. 2020;6(10):5399–5430. 33320556 10.1021/acsbiomaterials.0c00528

[16] Zhu Y, Zhang X, Chang G, Deng S, Chan HF. Bioactive glass in tissue regeneration: Unveiling recent advances in regenerative strategies and applications. Adv Mater. 2024; Article e2312964. 39014919 10.1002/adma.202312964 PMC11733714

[17] Rahaman MN, Day DE, Bal BS, Fu Q, Jung SB, Bonewald LF, Tomsia AP. Bioactive glass in tissue engineering. Acta Biomater. 2011;7(6):2355–2373. 21421084 10.1016/j.actbio.2011.03.016 PMC3085647

[18] Jones JR. Review of bioactive glass: From hench to hybrids. Acta Biomater. 2013;9(1):4457–4486. 22922331 10.1016/j.actbio.2012.08.023

[19] Sergi R, Bellucci D, Salvatori R, Maisetta G, Batoni G, Cannillo V. Zinc containing bioactive glasses with ultra-high crystallization temperature, good biological performance and antibacterial effects. Mater Sci Eng C Mater Biol Appl. 2019;104: Article 109910. 31500031 10.1016/j.msec.2019.109910

[20] Hench LL, Wilson J. *An Introduction to bioceramics*. Singapore: World Scientific; 1993.

[21] Kascholke C, Hendrikx S, Flath T, Kuzmenka D, Dorfler HM, Schumann D, Gressenbuch M, Schulze FP, Schulz-Siegmund M, Hacker MC. Biodegradable and adjustable sol-gel glass based hybrid scaffolds from multi-armed oligomeric building blocks. Acta Biomater. 2017;63:336–349. 28927930 10.1016/j.actbio.2017.09.024

[22] Brown RF, Day DE, Day TE, Jung S, Rahaman MN, Fu Q. Growth and differentiation of osteoblastic cells on 13-93 bioactive glass fibers and scaffolds. Acta Biomater. 2008;4(2):387–396. 17768097 10.1016/j.actbio.2007.07.006

[23] Dang W, Wang X, Li J, Deng C, Liu Y, Yao Q, Wang L, Chang J, Wu C. 3D printing of Mo-containing scaffolds with activated anabolic responses and bi-lineage bioactivities. Theranostics. 2018;8(16):4372–4392. 30214627 10.7150/thno.27088 PMC6134938

[24] Wu C, Zhou Y, Fan W, Han P, Chang J, Yuen J, Zhang M, Xiao Y. Hypoxia-mimicking mesoporous bioactive glass scaffolds with controllable cobalt ion release for bone tissue engineering. Biomaterials. 2012;33(7):2076–2085. 22177618 10.1016/j.biomaterials.2011.11.042

[25] Hu H, Tang Y, Pang L, Lin C, Huang W, Wang D, Jia W. Angiogenesis and full-thickness wound healing efficiency of a copper-doped borate bioactive glass/poly(lactic-co-glycolic acid) dressing loaded with vitamin E in vivo and in vitro. ACS Appl Mater Interfaces. 2018;10(27):22939–22950. 29924595 10.1021/acsami.8b04903

[26] Mountjoy G. Comment on ‘Bond volumes in crystals and glasses and a study of the germanate anomaly’ by H.-J. Weber [J. Non-Cryst. Solids 243(1999) 220]. J Non-Cryst Solids. 2003;324(1-2):177–178.

[27] Hench LL, Paschall HA. Direct chemical bond of bioactive glass-ceramic materials to bone and muscle. J Biomed Mater Res. 1973;7(3):25–42. 4123968 10.1002/jbm.820070304

[28] Ducheyne P. Bioceramics: Material characteristics versus in vivo behavior. J Biomed Mater Res. 1987;21(A2 Suppl):219–236. 3624287

[29] Lu HH, El-Amin SF, Scott KD, Laurencin CT. Three-dimensional, bioactive, biodegradable, polymer-bioactive glass composite scaffolds with improved mechanical properties support collagen synthesis and mineralization of human osteoblast-like cells. J Biomed Mater Res A. 2003;64A(3):465–474. 10.1002/jbm.a.10399 12579560

[30] Francis L, Meng D, Knowles JC, Roy I, Boccaccini AR. Multi-functional P(3HB) microsphere/45S5 Bioglass-based composite scaffolds for bone tissue engineering. Acta Biomater. 2010;6(7):2773–2786. 20056174 10.1016/j.actbio.2009.12.054

[31] Chandrasekar AR, Merino E, Pakseresht A, Galusek D, Duran A, Castro Y. Influence of polyols on the in vitro biodegradation and bioactivity of 58S bioactive sol-gel coatings on AZ31B magnesium alloys. Polymers. 2023;15(5):1273. 36904514 10.3390/polym15051273 PMC10007392

[32] Huang WH, Rahaman MN, Day DE, Li YD. Mechanisms for converting bioactive silicate, borate, and borosilicate glasses to hydroxyapatite in dilute phosphate solution. Phys Chem Glass B. 2006;47(6):647–658.

[33] Ning J, Yao A, Wang DP, Huang WH, Fu HL, Liu X, Jiang XQ, Zhang XL. Synthesis and in vitro bioactivity of a borate-based bioglass. Mater Lett. 2007;61(30):5223–5226.

[34] Li H, Li B, Lv D, Li W, Lu Y, Luo G. Biomaterials releasing drug responsively to promote wound healing via regulation of pathological microenvironment. Adv Drug Deliv Rev. 2023;196: Article 114778. 36931347 10.1016/j.addr.2023.114778

[35] Maeno S, Niki Y, Matsumoto H, Morioka H, Yatabe T, Funayama A, Toyama Y, Taguchi T, Tanaka J. The effect of calcium ion concentration on osteoblast viability, proliferation and differentiation in monolayer and 3D culture. Biomaterials. 2005;26(23):4847–4855. 15763264 10.1016/j.biomaterials.2005.01.006

[36] Josipovic I, Fork C, Preussner J, Prior KK, Iloska D, Vasconez AE, Labocha S, Angioni C, Thomas D, Os NF, et al. PAFAH1B1 and the lncRNA maintain an angiogenic phenotype in human endothelial cells. Acta Physiol. 2016;218(1):13–27. 10.1111/apha.12700 27124368

[37] Bao F, Pei G, Wu ZC, Zhuang H, Zhang ZWB, Huan ZG, Wu CT, Chang J. Bioactive self-pumping composite wound dressings with micropore array modified Janus membrane for enhanced diabetic wound healing. Adv Funct Mater. 2020;30(49):2005422.

[38] Pan HB, Zhao XL, Zhang X, Zhang KB, Li LC, Li ZY, Lam WM, Lu WW, Wang DP, Huang WH, et al. Strontium borate glass: Potential biomaterial for bone regeneration. J R Soc Interface. 2010;7(48):1025–1031. 20031984 10.1098/rsif.2009.0504 PMC2880081

[39] Liu J, Rawlinson SC, Hill RG, Fortune F. Strontium-substituted bioactive glasses in vitro osteogenic and antibacterial effects. Dent Mater. 2016;32(3):412–422. 26777094 10.1016/j.dental.2015.12.013

[40] Lapa A, Cresswell M, Campbell I, Jackson P, Goldmann WH, Detsch R, Parsons A, Ahmed I, Boccaccini AR. Ga and Ce ion-doped phosphate glass fibres with antibacterial properties and their composite for wound healing applications(vol 7, \pg 6981, 2019). J Mater Chem B. 2019;7(45):7246–7246. 31624824 10.1039/c9tb00820a

[41] Zheng K, Balasubramanian P, Paterson TE, Stein R, MacNeil S, Fiorilli S, Vitale-Brovarone C, Shepherd J, Boccaccini AR. Ag modified mesoporous bioactive glass nanoparticles for enhanced antibacterial activity in 3D infected skin model. Mater Sci Eng C Mater Biol Appl. 2019;103: Article 109764. 31349470 10.1016/j.msec.2019.109764

[42] Sharifi E, Sadati SA, Yousefiasl S, Sartorius R, Zafari M, Rezakhani L, Alizadeh M, Nazarzadeh Zare E, Omidghaemi S, Ghanavatinejad F, et al. Cell loaded hydrogel containing Ag-doped bioactive glass-ceramic nanoparticles as skin substitute: Antibacterial properties, immune response, and scarless cutaneous wound regeneration. Bioeng Transl Med. 2022;7(3): 10386. 10.1002/btm2.10386 PMC9471996 36176609

[43] Gerard C, Bordeleau LJ, Barralet J, Doillon CJ. The stimulation of angiogenesis and collagen deposition by copper. Biomaterials. 2010;31(5):824–831. 19854506 10.1016/j.biomaterials.2009.10.009

[44] Zhou Y, Han S, Xiao L, Han P, Wang S, He J, Chang J, Wu C, Xiao Y. Accelerated host angiogenesis and immune responses by ion release from mesoporous bioactive glass. J Mater Chem B. 2018;6(20):3274–3284. 32254385 10.1039/c8tb00683k

[45] Li Y, Xu T, Tu Z, Dai W, Xue Y, Tang C, Gao W, Mao C, Lei B, Lin C. Erratum: Bioactive antibacterial silica-based nanocomposites hydrogel scaffolds with high angiogenesis for promoting diabetic wound healing and skin repair: Erratum. Theranostics. 2022;12(10):4599–4600. 35832089 10.7150/thno.73263 PMC9254243

[46] Day RM, Boccaccini AR, Shurey S, Roether JA, Forbes A, Hench LL, Gabe SM. Assessment of polyglycolic acid mesh and bioactive glass for soft-tissue engineering scaffolds. Biomaterials. 2004;25(27):5857–5866. 15172498 10.1016/j.biomaterials.2004.01.043

[47] Varmette EA, Nowalk JR, Flick LM, Hall MM. Abrogation of the inflammatory response in LPS-stimulated RAW 264.7 murine macrophages by Zn- and cu-doped bioactive sol-gel glasses. J Biomed Mater Res A. 2009;90A(2):317–325. 10.1002/jbm.a.32098 18508353

[48] Brown RF, Rahaman MN, Dwilewicz AB, Huang W, Day DE, Li Y, Bal BS. Effect of borate glass composition on its conversion to hydroxyapatite and on the proliferation of MC3T3-E1 cells. J Biomed Mater Res A. 2009;88(2):392–400. 18306284 10.1002/jbm.a.31679

[49] Zhao SC, Li L, Wang H, Zhang YD, Cheng XG, Zhou N, Rahaman MN, Liu ZT, Huang WH, Zhang CQ. Wound dressings composed of copper-doped borate bioactive glass microfibers stimulate angiogenesis and heal full-thickness skin defects in a rodent model. Biomaterials. 2015;53:379–391. 25890736 10.1016/j.biomaterials.2015.02.112

[50] Lin C, Mao C, Zhang J, Li Y, Chen X. Healing effect of bioactive glass ointment on full-thickness skin wounds. Biomed Mater. 2012;7(4): Article 045017. 22736113 10.1088/1748-6041/7/4/045017

[51] Homaeigohar S, Li M, Boccaccini AR. Bioactive glass-based fibrous wound dressings. Burns Trauma. 2022;10:tkac038. 36196303 10.1093/burnst/tkac038 PMC9519693

[52] Hong Y, Chen X, Jing X, Fan H, Guo B, Gu Z, Zhang X. Preparation, bioactivity, and drug release of hierarchical nanoporous bioactive glass ultrathin fibers. Adv Mater. 2010;22(6):754–758. 20217784 10.1002/adma.200901656

[53] Li R, Clark AE, Hench LL. An investigation of bioactive glass powders by sol-gel processing. J Appl Biomater. 1991;2(4):231–239. 10171144 10.1002/jab.770020403

[54] Zeng Q, Han Y, Li H, Chang J. Design of a thermosensitive bioglass/agarose-alginate composite hydrogel for chronic wound healing. J Mater Chem B. 2015;3(45):8856–8864. 32263479 10.1039/c5tb01758k

[55] Zhou Y, Gao L, Peng J, Xing M, Han Y, Wang X, Xu Y, Chang J. Bioglass activated albumin hydrogels for wound healing. Adv Healthc Mater. 2018;7(16): Article e1800144. 29845777 10.1002/adhm.201800144

[56] Rodrigues M, Kosaric N, Bonham CA, Gurtner GC. Wound healing: A cellular perspective. Physiol Rev. 2019;99(1):665–706. 30475656 10.1152/physrev.00067.2017 PMC6442927

[57] Portou MJ, Baker D, Abraham D, Tsui J. The innate immune system, toll-like receptors and dermal wound healing: A review. Vasc Pharmacol. 2015;71:31–36. 10.1016/j.vph.2015.02.007 25869514

[58] Gurtner GC, Werner S, Barrandon Y, Longaker MT. Wound repair and regeneration. Nature. 2008;453(7193):314–321. 18480812 10.1038/nature07039

[59] Kolaczkowska E, Kubes P. Neutrophil recruitment and function in health and inflammation. Nat Rev Immunol. 2013;13(3):159–175. 23435331 10.1038/nri3399

[60] Brinkmann V, Reichard U, Goosmann C, Fauler B, Uhlemann Y, Weiss DS, Weinrauch Y, Zychlinsky A. Neutrophil extracellular traps kill bacteria. Science. 2004;303(5663):1532–1535. 15001782 10.1126/science.1092385

[61] Rosales C. Neutrophils at the crossroads of innate and adaptive immunity. J Leukoc Biol. 2020;108(1):377–396. 32202340 10.1002/JLB.4MIR0220-574RR

[62] Butin-Israeli V, Bui TM, Wiesolek HL, Mascarenhas L, Lee JJ, Mehl LC, Knutson KR, Adam SA, Goldman RD, Beyder A, et al. Neutrophil-induced genomic instability impedes resolution of inflammation and wound healing. J Clin Invest. 2019;129(2):712–726. 30640176 10.1172/JCI122085 PMC6355304

[63] Maitz MF, Gabriel E, Franke RP. Influence of bioactive glasses on the respiratory burst metabolism of polymorphonuclear neutrophils. Biomed Tech. 1999;44(6):172–175. 10.1515/bmte.1999.44.6.172 10427913

[64] Lindfors NC, Klockars M. Immunoglobulin enhances the bioactive-glass-induced chemiluminescence response of human polymorphonuclear leukocytes. J Biomed Mater Res. 2001;55(4):613–617. 11288090 10.1002/1097-4636(20010615)55:4<613::aid-jbm1055>3.0.co;2-h

[65] Terkeltaub RA, Santoro DA, Mandel G, Mandel N. Serum and plasma inhibit neutrophil stimulation by hydroxyapatite crystals—Evidence that serum alpha-2-Hs glycoprotein is a potent and specific crystal-bound inhibitor. Arthritis Rheum. 1988;31(9):1081–1089. 2844196 10.1002/art.1780310901

[66] Mahdavian Delavary B, van der Veer WM, van Egmond M, Niessen FB, Beelen RH. Macrophages in skin injury and repair. Immunobiology. 2011;216(7):753–762. 21281986 10.1016/j.imbio.2011.01.001

[67] Boniakowski AE, Kimball AS, Jacobs BN, Kunkel SL, Gallagher KA. Macrophage-mediated inflammation in normal and diabetic wound healing. J Immunol. 2017;199(1):17–24. 28630109 10.4049/jimmunol.1700223

[68] Roszer T. Understanding the mysterious M2 macrophage through activation markers and effector mechanisms. Mediat Inflamm. 2015;2015:816460. 10.1155/2015/816460 PMC4452191 26089604

[69] Smigiel KS, Parks WC. Macrophages, wound healing, and fibrosis: Recent insights. Curr Rheumatol Rep. 2018;20(4):17. 29550962 10.1007/s11926-018-0725-5

[70] Xiaojie W, Banda J, Qi H, Chang AK, Bwalya C, Chao L, Li X. Scarless wound healing: Current insights from the perspectives of TGF-beta, KGF-1, and KGF-2. Cytokine Growth Factor Rev. 2022;66:26–37. 35690568 10.1016/j.cytogfr.2022.03.001

[71] Meng T, He D, Han Z, Shi R, Wang Y, Ren B, Zhang C, Mao Z, Luo G, Den J. Nanomaterial-based repurposing of macrophage metabolism and its applications. Nanomicro Lett. 2024;16(1):246. 39007981 10.1007/s40820-024-01455-9 PMC11250772

[72] Bosetti M, Hench L, Cannas M. Interaction of bioactive glasses with peritoneal macrophages and monocytes. J Biomed Mater Res. 2002;60(1):79–85. 11835162 10.1002/jbm.1282

[73] Day RM, Boccaccini AR. Effect of particulate bioactive glasses on human macrophages and monocytes. J Biomed Mater Res A. 2005;73A(1):73–79. 10.1002/jbm.a.30262 15714504

[74] Barrak FN, Li SW, Mohammed AA, Myant C, Jones JR. Anti-inflammatory properties of S53P4 bioactive glass implant material. J Dent. 2022;127:104296. 36116542 10.1016/j.jdent.2022.104296

[75] Dong X, Chang J, Li HY. Bioglass promotes wound healing through modulating the paracrine effects between macrophages and repairing cells. J Mater Chem B. 2017;5(26):5240–5250. 32264109 10.1039/c7tb01211j

[76] Chen D, Liang ZT, Su ZK, Huang JY, Pi YX, Ouyang YT, Luo T, Guo L. Selenium-doped mesoporous bioactive glass regulates macrophage metabolism and polarization by scavenging ROS and promotes bone regeneration. ACS Appl Mater Interfaces. 2023;15(29):34378–34396. 37404000 10.1021/acsami.3c03446

[77] Chen C, Tang Y, Zhu X, Yang J, Liu Z, Chen Y, Wang J, Shang R, Zheng W, Zhang X, et al. P311 promotes IL-4 receptor–mediated M2 polarization of macrophages to enhance angiogenesis for efficient skin wound healing. J Invest Dermatol. 2023;143(4):648–660 e6. 36309321 10.1016/j.jid.2022.09.659

[78] Zhang L, Niu W, Lin Y, Ma J, Leng T, Cheng W, Wang Y, Wang M, Ning J, Yang S, et al. Multifunctional antibacterial bioactive nanoglass hydrogel for normal and MRSA infected wound repair. J Nanobiotechnology. 2023;21(1):162. 37211601 10.1186/s12951-023-01929-9 PMC10200057

[79] Zhang SX, Zhao LL, Chen ZS, Zhang LY, Li LC, Zhao MG, Yan LP, Liao LQ, Zhang C, Wu ZY. Macrophage-targeting bioactive glass nanoparticles for the treatment of intracellular infection and subcutaneous abscess. Biomater Sci. 2022;10(22):6535–6548. 36205236 10.1039/d2bm01117d

[80] Bussone G. Subjectivity in primary headaches: Insight the causes. Neurol Sci. 2017;38:S1–S2. 10.1007/s10072-017-2949-y 28527092

[81] Seki E, De Minicis S, Österreicher CH, Kluwe J, Osawa Y, Brenner DA, Schwabe RF. TLR4 enhances TGF-β signaling and hepatic fibrosis. Nat Med. 2007;13(11):1324–1332. 17952090 10.1038/nm1663

[82] Szabo G, Mandrekar P, Dolganiuc A. Innate immune response and hepatic inflammation. Semin Liver Dis. 2007;27(4):339–350. 17979071 10.1055/s-2007-991511

[83] Moretti L, Stalfort J, Barker TH, Abebayehu D. The interplay of fibroblasts, the extracellular matrix, and inflammation in scar formation. J Biol Chem. 2022;298(2): Article 101530. 34953859 10.1016/j.jbc.2021.101530 PMC8784641

[84] Chen C, Yang J, Shang R, Tang Y, Cai X, Chen Y, Liu Z, Hu W, Zhang W, Zhang X, et al. Orchestration of macrophage polarization dynamics by fibroblast-secreted exosomes during skin wound healing. J Invest Dermatol. 2025;145(1):171–184.e6. 38838771 10.1016/j.jid.2024.05.007

[85] Keshaw H, Forbes A, Day RM. Release of angiogenic growth factors from cells encapsulated in alginate beads with bioactive glass. Biomaterials. 2005;26(19):4171–4179. 15664644 10.1016/j.biomaterials.2004.10.021

[86] Zhang M, Fan Z, Zhang J, Yang Y, Huang C, Zhang W, Ding D, Liu G, Cheng N. Multifunctional chitosan/alginate hydrogel incorporated with bioactive glass nanocomposites enabling photothermal and nitric oxide release activities for bacteria-infected wound healing. Int J Biol Macromol. 2023;232: Article 123445. 36709818 10.1016/j.ijbiomac.2023.123445

[87] Yu H, Peng J, Xu Y, Chang J, Li H. Bioglass activated skin tissue engineering constructs for wound healing. ACS Appl Mater Interfaces. 2016;8(1):703–715. 26684719 10.1021/acsami.5b09853

[88] Day RM. Bioactive glass stimulates the secretion of angiogenic growth factors and angiogenesis in vitro. Tissue Eng. 2005;11(5-6):768–777. 15998217 10.1089/ten.2005.11.768

[89] Wang X, Cheng F, Liu J, Smatt JH, Gepperth D, Lastusaari M, Xu C, Hupa L. Biocomposites of copper-containing mesoporous bioactive glass and nanofibrillated cellulose: Biocompatibility and angiogenic promotion in chronic wound healing application. Acta Biomater. 2016;46:286–298. 27646503 10.1016/j.actbio.2016.09.021

[90] Kohoolat G, Alizadeh P, Motesadi Zarandi F, Rezaeipour Y. A ternary composite hydrogel based on sodium alginate, carboxymethyl cellulose and copper-doped 58S bioactive glass promotes cutaneous wound healing in vitro and in vivo. Int J Biol Macromol. 2024;259(Part 2): Article 129260. 38199544 10.1016/j.ijbiomac.2024.129260

[91] Xie W, Chen X, Miao G, Tang J, Fu X. Regulation of cellular behaviors of fibroblasts related to wound healing by sol-gel derived bioactive glass particles. J Biomed Mater Res A. 2016;104(10):2420–2429. 27177533 10.1002/jbm.a.35782

[92] Yu YF, Wang CC, Fu QQ, Wan Y, Yu AX. Multi-crosslinked hydrogel built with hyaluronic acid-tyramine, thiolated glycol chitosan and copper-doped bioglass nanoparticles for expediting wound healing. Carbohyd Polym. 2024;327:121635. 10.1016/j.carbpol.2023.121635 38171654

[93] Martin P. Wound healing—Aiming for perfect skin regeneration. Science. 1997;276(5309):75–81. 9082989 10.1126/science.276.5309.75

[94] Hum J, Boccaccini AR. Bioactive glasses as carriers for bioactive molecules and therapeutic drugs: A review. J Mater Sci Mater Med. 2012;23(10):2317–2333. 22361998 10.1007/s10856-012-4580-z

[95] Tellado SF, Delgado JA, Poh SPP, Zhang W, García-Vallés M, Martínez S, Gorustovich A, Morejón L, van Griensven M, Balmayor ER. Phosphorous pentoxide-free bioactive glass exhibits dose-dependent angiogenic and osteogenic capacities which are retained in glass polymeric composite scaffolds. Biomater Sci. 2021;9(23):7876–7894. 34676835 10.1039/d1bm01311d

[96] Bellucci D, Braccini S, Chiellini F, Balasubramanian P, Boccaccini AR, Cannillo V. Bioactive glasses and glass-ceramics versus hydroxyapatite: Comparison of angiogenic potential and biological responsiveness. J Biomed Mater Res A. 2019;107(12):2601–2609. 31376313 10.1002/jbm.a.36766

[97] Schumacher M, Habibovic P, van Rijt S. Peptide-modified nano-bioactive glass for targeted immobilization of native VEGF. ACS Appl Mater Interfaces. 2022;14(4):4959–4968. 35041377 10.1021/acsami.1c21378 PMC8815037

[98] Li H, He J, Yu H, Green CR, Chang J. Bioglass promotes wound healing by affecting gap junction connexin 43 mediated endothelial cell behavior. Biomaterials. 2016;84:64–75. 26821121 10.1016/j.biomaterials.2016.01.033

[99] Kermani F, Nazarnezhad S, Mollaei Z, Mollazadeh S, Ebrahimzadeh-Bideskan A, Askari VR, Oskuee RK, Moradi A, Hosseini SA, Azari Z, et al. Zinc- and copper-doped mesoporous borate bioactive glasses: Promising additives for potential use in skin wound healing applications. Int J Mol Sci. 2023;24(2):1304. 36674818 10.3390/ijms24021304 PMC9861609

[100] Yu Y, Yang B, Tian D, Liu J, Yu A, Wan Y. Thiolated hyaluronic acid/silk fibroin dual-network hydrogel incorporated with bioglass nanoparticles for wound healing. Carbohydr Polym. 2022;288: Article 119334. 35450620 10.1016/j.carbpol.2022.119334

[101] Li J, Zhai D, Lv F, Yu Q, Ma H, Yin J, Yi Z, Liu M, Chang J, Wu C. Preparation of copper-containing bioactive glass/eggshell membrane nanocomposites for improving angiogenesis, antibacterial activity and wound healing. Acta Biomater. 2016;36:254–266. 26965395 10.1016/j.actbio.2016.03.011

[102] Solanki AK, Lali FV, Autefage H, Agarwal S, Nommeots-Nomm A, Metcalfe AD, Stevens MM, Jones JR. Bioactive glasses and electrospun composites that release cobalt to stimulate the HIF pathway for wound healing applications. Biomater Res. 2021;25(1):1. 33451366 10.1186/s40824-020-00202-6 PMC7811269

[103] Tang F, Li J, Xie W, Mo Y, Ouyang L, Zhao F, Fu X, Chen X. Bioactive glass promotes the barrier functional behaviors of keratinocytes and improves the re-epithelialization in wound healing in diabetic rats. Bioact Mater. 2021;6(10):3496–3506. 33817423 10.1016/j.bioactmat.2021.02.041 PMC7988492

[104] Warren R, Chestnut MH, Wong TK, Otte TE, Lammers KM, Meili ML. An improved method for the isolation and cultivation of human scalp dermal papilla cells: Maintenance of extracellular matrix. Ann N Y Acad Sci. 1991;642:436–438. 1809097 10.1111/j.1749-6632.1991.tb24409.x

[105] Huang SH, Lin YN, Lee SS, Chai CY, Chang HW, Lin TM, Lai CS, Lin SD. New adipose tissue formation by human adipose-derived stem cells with hyaluronic acid gel in immunodeficient mice. Int J Med Sci. 2015;12(2):154–162. 25589892 10.7150/ijms.9964 PMC4293181

[106] Choi EW, Seo MK, Woo EY, Kim SH, Park EJ, Kim S. Exosomes from human adipose-derived stem cells promote proliferation and migration of skin fibroblasts. Exp Dermatol. 2018;27(10):1170–1172. 28940813 10.1111/exd.13451

[107] Mizuno H, Tobita M, Uysal AC. Concise review: Adipose-derived stem cells as a novel tool for future regenerative medicine. Stem Cells. 2012;30(5):804–810. 22415904 10.1002/stem.1076

[108] Marfia G, Navone SE, Di Vito C, Ughi N, Tabano S, Miozzo M, Tremolada C, Bolla G, Crotti C, Ingegnoli F, et al. Mesenchymal stem cells: Potential for therapy and treatment of chronic non-healing skin wounds. Organogenesis. 2015;11(4):183–206. 26652928 10.1080/15476278.2015.1126018 PMC4879897

[109] Duran RCD, González-Garza MT, Cardenas-Lopez A, Chavez-Castilla L, Cruz-Vega DE, Moreno-Cuevas JE. Age-related yield of adipose-derived stem cells bearing the low-affinity nerve growth factor receptor. Stem Cells Int. 2013;2013:372164. 24376462 10.1155/2013/372164 PMC3859201

[110] Ferreira ADF, Gomes DA. Stem cell extracellular vesicles in skin repair. Bioengineering. 2018;6(1):4. 30598033 10.3390/bioengineering6010004 PMC6466099

[111] Cappuzzello C, Doni A, Dander E, Pasqualini F, Nebuloni M, Bottazzi B, Mantovani A, Biondi A, Garlanda C, D’Amico G. Mesenchymal stromal cell-derived PTX3 promotes wound healing via fibrin remodeling. J Invest Dermatol. 2016;136(1):293–300. 26763449 10.1038/JID.2015.346

[112] Ding JY, Chen MJ, Wu LF, Shu GF, Fang SJ, Li ZY, Chu XR, Li XK, Wang ZG, Ji JS. Mesenchymal stem cell-derived extracellular vesicles in skin wound healing: Roles, opportunities and challenges. Mil Med Res. 2023;10(1):36. 37587531 10.1186/s40779-023-00472-w PMC10433599

[113] Seo YS, Ko IO, Park H, Jeong YJ, Park JA, Kim KS, Park MJ, Lee HJ. Radiation-induced changes in tumor vessels and microenvironment contribute to therapeutic resistance in glioblastoma. Front Oncol. 2019;9:1259. 31803626 10.3389/fonc.2019.01259 PMC6873882

[114] De Melo N, Murrell L, Islam MT, Titman JJ, Macri-Pellizzeri L, Ahmed I, Sottile V. Tailoring pyro-and orthophosphate species to enhance stem cell adhesion to phosphate glasses. Int J Mol Sci. 2021;22(2):837. 33467686 10.3390/ijms22020837 PMC7829838

[115] Xu H, Zhu Y, Hsiao AW, Xu J, Tong W, Chang L, Zhang X, Chen YF, Li J, Chen W, et al. Bioactive glass-elicited stem cell-derived extracellular vesicles regulate M2 macrophage polarization and angiogenesis to improve tendon regeneration and functional recovery. Biomaterials. 2023;294: Article 121998. 36641814 10.1016/j.biomaterials.2023.121998

[116] Salinas AJ, Shruti S, Malavasi G, Menabue L, Vallet-Regi M. Substitutions of cerium, gallium and zinc in ordered mesoporous bioactive glasses. Acta Biomater. 2011;7(9):3452–3458. 21672640 10.1016/j.actbio.2011.05.033

[117] Roy P, Saha R, Chakraborty J. A novel composition of bioactive glass with potent haemostatic action and antibacterial competence. Ceram Int. 2023;49(4):6389–6400.

[118] Gordon PR, Woodruff CW, Anderson HL, Odell BL. Effect of acute zinc deprivation on plasma zinc and platelet-aggregation in adult males. Am J Clin Nutr. 1982;35(1):113–119. 6801956 10.1093/ajcn/35.1.113

[119] Wang Y, Luo M, Li T, Xie C, Li S, Lei B. Multi-layer-structured bioactive glass nanopowder for multistage-stimulated hemostasis and wound repair. Bioact Mater. 2023;25:319–332. 36844363 10.1016/j.bioactmat.2023.01.019 PMC9946820

[120] Powers JG, Higham C, Broussard K, Phillips TJ. Wound healing and treating wounds: Chronic wound care and management. J Am Acad Dermatol. 2016;74(4):607–625. 26979353 10.1016/j.jaad.2015.08.070

[121] Werdin F, Tenenhaus M, Rennekampff HO. Chronic wound care. Lancet. 2008;372(9653):1860–1862. 19041788 10.1016/S0140-6736(08)61793-6

[122] Solanki AK, Autefage H, Rodriguez AR, Agarwal S, Penide J, Mahat M, Whittaker T, Nommeots-Nomm A, Littmann E, Payne DJ, et al. Cobalt containing glass fibres and their synergistic effect on the HIF-1 pathway for wound healing applications. Front Bioeng Biotechnol. 2023;11:1125060. 36970616 10.3389/fbioe.2023.1125060 PMC10036384

[123] Shirgill S, Poologasundarampillai G, Kuehne S, Jabbari S, Ward J. Investigating the antimicrobial effects of metal-doped bioactive glass fibres on chronic wound biofilms. *Tissue Eng Pt A*. 2023;**29**(13-14). 10.1016/j.bioflm.2023.100115 PMC10209705 37252225

[124] James GA, Swogger E, Wolcott R, Pulcini ED, Secor P, Sestrich J, Costerton JW, Stewart PS. Biofilms in chronic wounds. Wound Repair Regen. 2008;16(1):37–44. 18086294 10.1111/j.1524-475X.2007.00321.x

[125] Cerruti M, Greenspan D, Powers K. Effect of pH and ionic strength on the reactivity of Bioglass 45S5. Biomaterials. 2005;26(14):1665–1674. 15576140 10.1016/j.biomaterials.2004.07.009

[126] Hao ZM, Yang H, Meng YB. Dermlin and silver nanoparticles combined antibacterial dressing for skin wound repair. Sci Adv Mater. 2021;13(10):1945–1950.

[127] Feuchtinger J, Halfens R, Dassen T. Pressure ulcer risk assessment immediately after cardiac surgery—Does it make a difference? A comparison of three pressure ulcer risk assessment instruments within a cardiac surgery population. Nurs Crit Care. 2007;12(1):42–49. 17883663 10.1111/j.1478-5153.2006.00198.x

[128] Li ZY, Lin F, Thalib L, Chaboyer W. Global prevalence and incidence of pressure injuries in hospitalised adult patients: A systematic review and meta-analysis. Int J Nurs Stud. 2020;105:103546. 32113142 10.1016/j.ijnurstu.2020.103546

[129] Saibertová S, Pokorná A, Vasmanská S, Búrilová P, Müllerová N, Fiedlerová L, Svobodová D, Camprová P, Smelková G, Kubátová L. Evaluation of selected pressure ulcer management international guidelines (AGREE II Tool). Cesk Slov Neurol N. 2016;79:S40–S44.

[130] Norman G, Dumville JC, Moore ZE, Tanner J, Christie J, Goto S. Antibiotics and antiseptics for pressure ulcers. Cochrane Database Syst Rev. 2016;4(4):CD011586. 27040598 10.1002/14651858.CD011586.pub2 PMC6486293

[131] Moore ZE, Webster J. Dressings and topical agents for preventing pressure ulcers. Cochrane Database Syst Rev. 2018;12(12):CD009362. 30537080 10.1002/14651858.CD009362.pub3 PMC6517041

[132] Westby MJ, Dumville JC, Soares MO, Stubbs N, Norman G. Dressings and topical agents for treating pressure ulcers. Cochrane Database Syst Rev. 2017;6(6):CD011947. 28639707 10.1002/14651858.CD011947.pub2 PMC6481609

[133] Zhu YN, Zhang JM, Song JY, Yang J, Du Z, Zhao WQ, Guo HS, Wen CY, Li QS, Sui XJ, et al. A multifunctional pro-healing zwitterionic hydrogel for simultaneous optical monitoring of pH and glucose in diabetic wound treatment. Adv Funct Mater. 2020;30(6):1905493.

[134] Chen J, Liu YJ, Cheng GP, Guo JH, Du S, Qiu JM, Wang C, Li CC, Yang XF, Chen TK, et al. Tailored hydrogel delivering niobium carbide boosts ROS-scavenging and antimicrobial activities for diabetic wound healing. Small. 2022;18(27):2201300. 10.1002/smll.202201300 35678523

[135] Chen YH, Rao ZF, Liu YJ, Liu XS, Liu YF, Xu LJ, Wang ZQ, Guo JY, Zhang L, Dong YS, et al. Multifunctional injectable hydrogel loaded with cerium-containing bioactive glass nanoparticles for diabetic wound healing. Biomol Ther. 2021;11(5):702. 10.3390/biom11050702 PMC8151889 34066859

[136] Jiang YF, Wang XM, Xia L, Fu XB, Xu ZR, Ran XW, Yan L, Li Q, Mo ZH, Yan ZL, et al. A cohort study of diabetic patients and diabetic foot ulceration patients in China. Wound Repair Regen. 2015;23(2):222–230. 25682850 10.1111/wrr.12263

[137] Xie W, Fu X, Tang F, Mo Y, Cheng J, Wang H, Chen X. Dose-dependent modulation effects of bioactive glass particles on macrophages and diabetic wound healing. J Mater Chem B. 2019;7(6):940–952. 32255099 10.1039/c8tb02938e

[138] Lv F, Wang J, Xu P, Han Y, Ma H, Xu H, Chen S, Chang J, Ke Q, Liu M, et al. A conducive bioceramic/polymer composite biomaterial for diabetic wound healing. Acta Biomater. 2017;60:128–143. 28713016 10.1016/j.actbio.2017.07.020

[139] Galeano M, Altavilla D, Cucinotta D, Russo GT, Calo M, Bitto A, Marini H, Marini R, Adamo EB, Seminara P, et al. Recombinant human erythropoietin stimulates angiogenesis and wound healing in the genetically diabetic mouse. Diabetes. 2004;53(9):2509–2517. 15331568 10.2337/diabetes.53.9.2509

[140] Zhou X, Patel D, Sen S, Shanmugam V, Sidawy A, Mishra L, Nguyen BN. Poly-ADP-ribose polymerase inhibition enhances ischemic and diabetic wound healing by promoting angiogenesis. J Vasc Surg. 2017;65(4):1161–1169. 27288104 10.1016/j.jvs.2016.03.407

[141] Mao C, Lin C, Chen XF. Enhanced healing of full-thickness diabetic wounds using bioactive glass and Yunnan Baiyao ointments. J Wuhan Univ Technol. 2014;29(5):1063–1070.

[142] Li J, Lv F, Xu H, Zhang Y, Wang J, Yi Z, Yin J, Chang J, Wu C. A patterned nanocomposite membrane for high-efficiency healing of diabetic wound. J Mater Chem B. 2017;5(10):1926–1934. 32263946 10.1039/c7tb00124j

[143] Gao W, Jin W, Li Y, Wan L, Wang C, Lin C, Chen X, Lei B, Mao C. A highly bioactive bone extracellular matrix-biomimetic nanofibrous system with rapid angiogenesis promotes diabetic wound healing. J Mater Chem B. 2017;5(35):7285–7296. 32264178 10.1039/c7tb01484h

[144] Xiao SN, Zhao TF, Wang JK, Wang CG, Du JN, Ying LW, Lin JT, Zhang CH, Hu WL, Wang LN, et al. Gelatin methacrylate(GelMA)-based hydrogels for cell transplantation: An effective strategy for tissue engineering. Stem Cell Rev Rep. 2019;15(5):664–679. 31154619 10.1007/s12015-019-09893-4

[145] Kong LZ, Wu Z, Zhao HK, Cui HM, Shen J, Chang J, Li HY, He YH. Bioactive injectable hydrogels containing desferrioxamine and bioglass for diabetic wound healing. ACS Appl Mater Interfaces. 2018;10(36):30103–30114. 30113159 10.1021/acsami.8b09191

[146] Li YN, Xu TZ, Tu ZL, Dai WT, Xue YM, Tang CX, Gao WY, Mao C, Lei B, Lin C. Bioactive antibacterial silica-based nanocomposites hydrogel scaffolds with high angiogenesis for promoting diabetic wound healing and skin repair(vol 10, pg 4929, 2020). Theranostics. 2022;12(10):4599–4600. 35832089 10.7150/thno.73263 PMC9254243

[147] Shang S, Zhuang K, Chen J, Zhang M, Jiang S, Li W. A bioactive composite hydrogel dressing that promotes healing of both acute and chronic diabetic skin wounds. Bioact Mater. 2024;34:298–310. 38261910 10.1016/j.bioactmat.2023.12.026 PMC10796815

[148] Manjubaashini N, Bargavi P, Thomas NG, Krishnan N, Balakumar S. Chitosan bioactive glass scaffolds for subcutaneous implantation, toxicity assessment, and diabetic wound healing upon animal model. Int J Biol Macromol. 2024;256:128291. 38029901 10.1016/j.ijbiomac.2023.128291

[149] Chen YH, Rao ZF, Liu YJ, Liu XS, Liu YF, Xu LJ, Wang ZQ, Guo JY, Zhang L, Dong YS, et al. Correction: Chen et al. Multifunctional injectable hydrogel loaded with cerium-containing bioactive glass nanoparticles for diabetic wound healing. Biomol Ther. 2021;11(5):705. 10.3390/biom11050702 PMC8151889 34066859

[150] Liu Z, Wang F, Ren J, Qu X. A series of MOF/Ce-based nanozymes with dual enzyme-like activity disrupting biofilms and hindering recolonization of bacteria. Biomaterials. 2019;208:21–31. 30986610 10.1016/j.biomaterials.2019.04.007

[151] Hyslop PA, Hinshaw DB, Scraufstatter IU, Cochrane CG, Kunz S, Vosbeck K. Hydrogen-peroxide as a potent bacteriostatic antibiotic—Implications for host-defense. Free Radical Bio Med. 1995;19(1):31–37. 7635356 10.1016/0891-5849(95)00005-i

[152] Cho M, Hunt TK, Hussain MZ. Hydrogen peroxide stimulates macrophage vascular endothelial growth factor release. Am J Physiol Heart Circ Physiol. 2001;280(5):H2357–H2363. 11299242 10.1152/ajpheart.2001.280.5.H2357

[153] Xian DH, Song J, Yang LY, Xiong X, Lai R, Zhong JQ. Emerging roles of redox-mediated angiogenesis and oxidative stress in dermatoses. Oxidative Med Cell Longev. 2019;2019:2304018. 10.1155/2019/2304018 PMC6501144 31178954

[154] Huang J, Zheng Y, Niu H, Huang J, Zhang X, Chen J, Ma B, Wu C, Cao Y, Zhu Y. A multifunctional hydrogel for simultaneous visible H_2_O_2_ monitoring and accelerating diabetic wound healing. Adv Healthc Mater. 2024;13(3): Article e2302328. 37824839 10.1002/adhm.202302328

[155] Bargavi P, Balakumar S, Raghunandhakumar S. Multi-functional bandage—Bioactive glass/metal oxides/alginate composites based regenerative membrane facilitating re-epithelialization in diabetic wounds with sustained drug delivery and anti-bactericidal efficacy. Int J Biol Macromol. 2024;262(Pt 2): Article 130054. 38342258 10.1016/j.ijbiomac.2024.130054

[156] Sen CK. Human wounds and its burden: An updated compendium of estimates. Adv Wound Care. 2019;8(2):39–48. 10.1089/wound.2019.0946 PMC6389759 30809421

[157] Zavlin D, Chegireddy V, Boukovalas S, Nia AM, Branski LK, Friedman JD, Echo A. Multi-institutional analysis of independent predictors for burn mortality in the United States. Burns Trauma. 2018;6:24. 30151396 10.1186/s41038-018-0127-y PMC6103989

[158] Fayyazbakhsh F, Khayat MJ, Sadler C, Day D, Huang YW, Leu MC. 3D-printed hydrogels dressings with bioactive borate glass for continuous hydration and treatment of second-degree burns. Int J Bioprint. 2023;9(6):0118. 38516674 10.36922/ijb.0118 PMC10956508

[159] Monafo WW, Tandon SN, Ayvazian VH, Tuchschmidt J, Skinner AM, Deitz F. Cerium nitrate: A new topical antiseptic for extensive burns. Surgery. 1976;80(4):465–473. 135364

[160] Krishnan P, Frew Q, Green A, Martin R, Dziewulski P. Cause of death and correlation with autopsy findings in burns patients. Burns. 2013;39(4):583–588. 23137628 10.1016/j.burns.2012.09.017

[161] Gong Y, Peng Y, Luo X, Zhang C, Shi Y, Zhang Y, Deng J, Peng Y, Luo G, Li H. Different infection profiles and antimicrobial resistance patterns between burn ICU and common wards. Front Cell Infect Microbiol. 2021;11: Article 681731. 34277469 10.3389/fcimb.2021.681731 PMC8278283

[162] Kowal S, Kruger E, Bilir P, Holmes JH, Hickerson W, Foster K, Nystrom S, Sparks J, Iyer N, Bush K, et al. Cost-effectiveness of the use of autologous cell harvesting device compared to standard of care for treatment of severe burns in the United States. Adv Ther. 2019;36(7):1715–1729. 31065995 10.1007/s12325-019-00961-2 PMC6647544

[163] Barsoumian A, Sanchez CJ, Mende K, Tully CC, Beckius ML, Akers KS, Wenke JC, Murray CK. In vitro toxicity and activity of Dakin’s solution, mafenide acetate, and amphotericin B on filamentous fungi and human cells. J Orthop Trauma. 2013;27(8):428–436. 23287750 10.1097/BOT.0b013e3182830bf9

[164] Kargozar S, Mozafari M, Hamzehlou S, Baino F. Using bioactive glasses in the management of burns. Front Bioeng Biotechnol. 2019;7:62. 30984751 10.3389/fbioe.2019.00062 PMC6447657

[165] Prabhakaran HS, Hu D, He W, Luo G, Liou YC. Mitochondrial dysfunction and mitophagy: Crucial players in burn trauma and wound healing. Burns Trauma. 2023;11:tkad029. 37465279 10.1093/burnst/tkad029 PMC10350398

[166] He XT, Li X, Zhang M, Tian BM, Sun LJ, Bi CS, Deng DK, Zhou H, Qu HL, Wu C, et al. Role of molybdenum in material immunomodulation and periodontal wound healing: Targeting immunometabolism and mitochondrial function for macrophage modulation. Biomaterials. 2022;283: Article 121439. 35247634 10.1016/j.biomaterials.2022.121439

[167] Ju Q, Zenji T, Maçon ALB, Norris E, Poologasundarampillai G, Obata A, Jones JR, Kasuga T. Silver-doped calcium silicate sol-gel glasses with a cotton-wool-like structure for wound healing. Biomater Adv. 2022;134:112561. 35523641 10.1016/j.msec.2021.112561

[168] Kermani F, Sadidi H, Ahmadabadi A, Hoseini SJ, Tavousi SH, Rezapanah A, Nazarnezhad S, Hosseini SA, Mollazadeh S, Kargozar S. Modified sol-gel synthesis of mesoporous borate bioactive glasses for potential use in wound healing. Bioengineering. 2022;9(9):442. 36134988 10.3390/bioengineering9090442 PMC9495454

[169] Zhu XR, Kazemi A, Dong YQ, Pan Q, Jin PS, Cheng BA, Yang YO. Effectiveness of nano bioactive glass fiber loaded with platelet-rich plasma on thermal wound healing process in rats. J Biomed Nanotechnol. 2022;18(2):535–545. 35484761 10.1166/jbn.2022.3249

[170] Hoffman AS. Hydrogels for biomedical applications. Adv Drug Deliver Rev. 2012;64:18–23. 10.1016/s0169-409x(01)00239-3 11755703

[171] Shevchenko RV, Sibbons PD, Sharpe JR, James SE. Use of a novel porcine collagen paste as a dermal substitute in full-thickness wounds. Wound Repair Regen. 2008;16(2):198–207. 18318805 10.1111/j.1524-475X.2008.00360.x

[172] Wu S, Cheng X, Xu X, Wu J, Huang Z, Guo Z, He P, Zhou C, Li H. In vivo and in vitro evaluation of chitosan-modified bioactive glass paste for wound healing. J Mater Chem B. 2022;10(4):598–606. 34988576 10.1039/d1tb02083h

[173] Hu X, Wang G, Cheng D. Study on efficacy and safety of bioactive material--combest in treating burn. Zhongguo xiu fu chong jian wai ke za zhi. 2007;21(11):1216–1218. 18069479

[174] Wang ZC, Zhao WY, Cao Y, Liu YQ, Sun Q, Shi P, Cai JQ, Shen XZ, Tan WQ. The roles of inflammation in keloid and hypertrophic scars. Front Immunol. 2020;11: Article 603187. 33343575 10.3389/fimmu.2020.603187 PMC7746641

[175] Zhang Q, Shi L, He H, Liu X, Huang Y, Xu D, Yao M, Zhang N, Guo Y, Lu Y, et al. Down-regulating scar formation by microneedles directly via a mechanical communication pathway. ACS Nano. 2022;16(7):10163–10178. 35617518 10.1021/acsnano.1c11016 PMC9331171

[176] Zhu Z, Ding J, Ma Z, Iwashina T, Tredget EE. Systemic depletion of macrophages in the subacute phase of wound healing reduces hypertrophic scar formation. Wound Repair Regen. 2016;24(4):644–656. 27169512 10.1111/wrr.12442

[177] Xiang Y, Fan B, Shang P, Ding R, Du J, Zhu T, Zhang H, Yan X. VR23 and bisdemethoxycurcumin enhanced nanofiber niche with durable bidirectional functions for promoting wound repair and inhibiting scar formation. Small Methods. 2024;8(10):e2400273. 38733258 10.1002/smtd.202400273

[178] Xi Y, Ge J, Wang M, Chen M, Niu W, Cheng W, Xue Y, Lin C, Lei B. Bioactive anti-inflammatory, antibacterial, antioxidative silicon-based nanofibrous dressing enables cutaneous tumor photothermo-chemo therapy and infection-induced wound healing. ACS Nano. 2020;14(3):2904–2916. 32031782 10.1021/acsnano.9b07173

[179] Yuan C, Zhang D, Tang Y, Guo Z, Lin K, Yu Y, Li J, Cai Q. Fibrous dressing containing bioactive glass with combined chemotherapy and wound healing promotion for post-surgical treatment of melanoma. Biomater Adv. 2023;149: Article 213387. 36990026 10.1016/j.bioadv.2023.213387

[180] Chen M, Wang M, Niu W, Cheng W, Guo Y, Wang Y, Luo M, Xie C, Leng T, Zhang X, et al. Multifunctional protein-decorated bioactive glass nanoparticles for tumor-specific therapy and bioimaging in vitro and in vivo. ACS Appl Mater Interfaces. 2021;13(13):14985–14994. 33779130 10.1021/acsami.1c01337

[181] Niu W, Chen M, Guo Y, Wang M, Luo M, Cheng W, Wang Y, Lei B. A multifunctional bioactive glass-ceramic nanodrug for post-surgical infection/cancer therapy-tissue regeneration. ACS Nano. 2021;15(9):14323–14337. 34491737 10.1021/acsnano.1c03214

[182] Huang H, Wang XR, Wang WL, Qu XY, Song XJ, Zhang YW, Zhong LP, Yang DP, Dong XC, Zhao YX. Injectable hydrogel for postoperative synergistic photothermal-chemodynamic tumor and anti-infection therapy. Biomaterials. 2022;280:121289. 34861512 10.1016/j.biomaterials.2021.121289

[183] Liu XY, Shen MF, Bing TJ, Zhang XY, Li YF, Cai Q, Yang XP, Yu YJ. A bioactive injectable hydrogel regulates tumor metastasis and wound healing for melanoma via NIR-light triggered hyperthermia. Adv Sci. 2024;11(26):2402208. 10.1002/advs.202402208 PMC11234446 38704692

