# Supplementary material for: Bioactive Glasses: Advancing Skin Tissue Repair through Multifunctional Mechanisms and Innovations
Source: Biomater Res. 2025 Jan 22;29:0134. doi: 10.34133/bmr.0134 (PMC11751205; doi:10.34133/bmr.0134)
Supplement: Supplementary 1 — Figs. S1 to S3 [file bmr.0134.f1.docx]

**Supplemental Material**


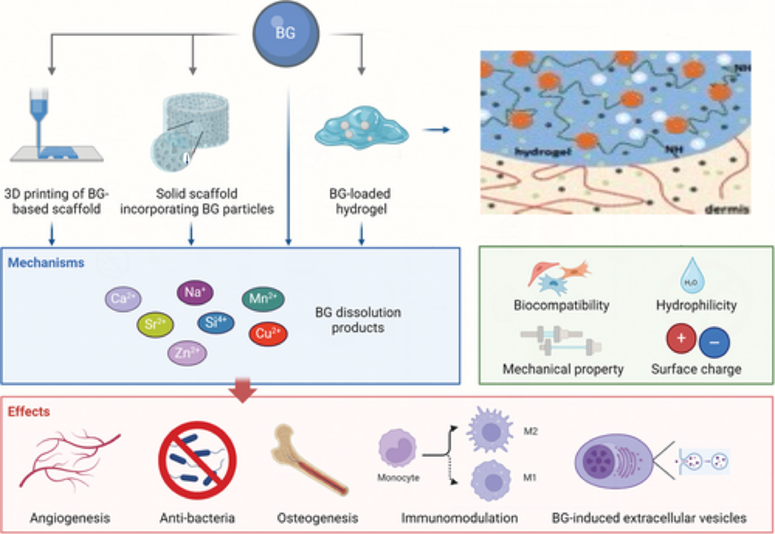


**Figure S1.**

BG improves its mechanical properties and biocompatibility by forming solid scaffolds based on 3D printing, microfibrous cottonlike composite scaffolds and hydrogels, and releases functional ions in the body to play a role in promoting angiogenesis, osteogenesis, immunomodulation, inhibiting bacterial growth, etc. (Adapted from [15][16])


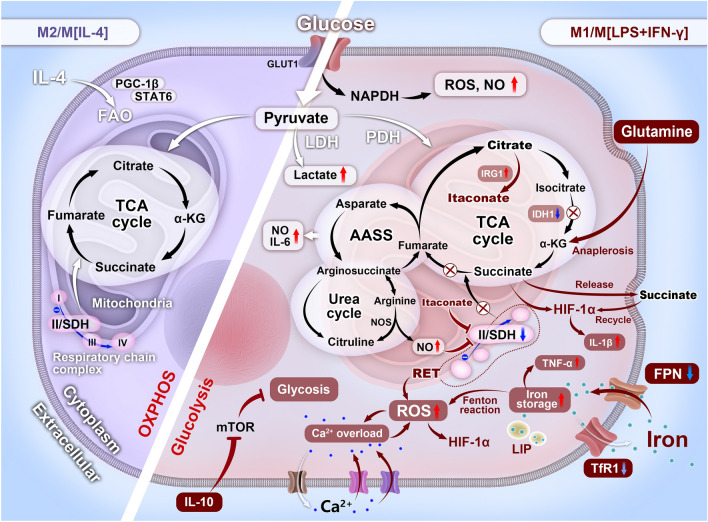


**Figure S2.**

Metabolic adaptation in M1 and M2 macrophages in response to environmental cues: an underlying target for macrophage theranostics. (Adapted from [72])


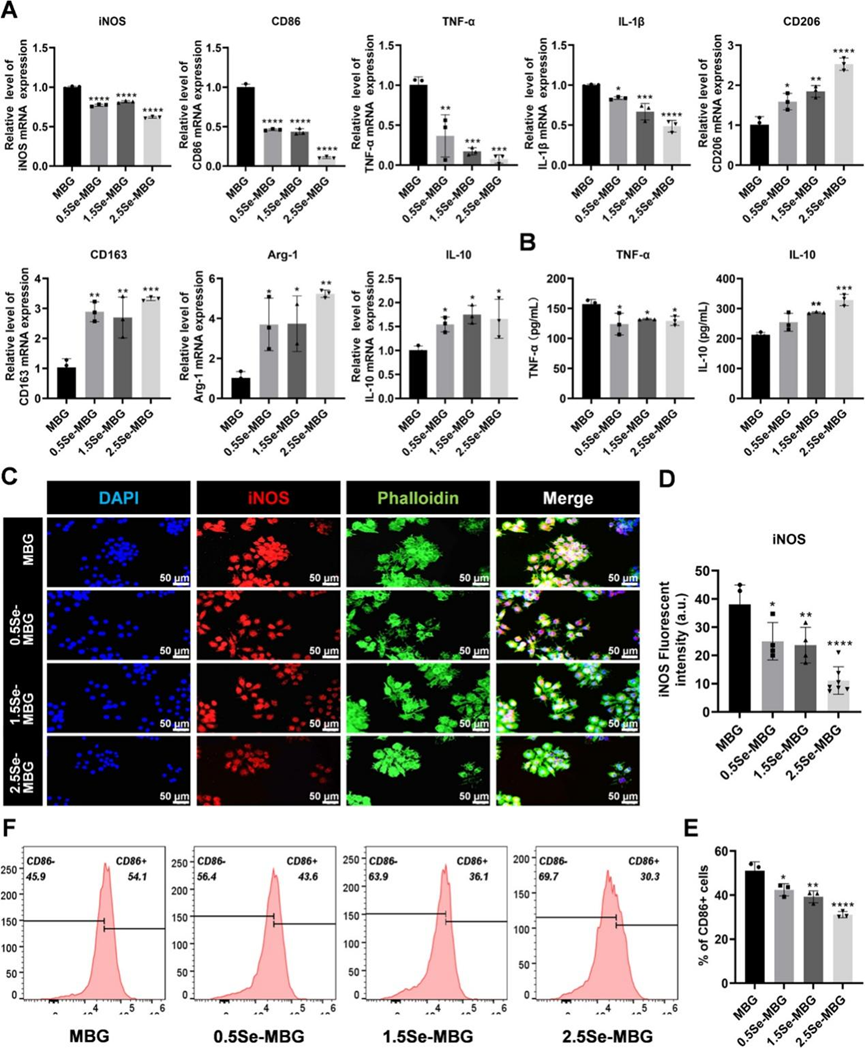


**Figure S3.**

Se-MBG extracts induce macrophage M2 polarization. (A) qRT-PCR analysis of M1 (iNOS, CD86, TNF-α, and IL-1β) and M2 (CD206, CD163, Arg-1, and IL-10) macrophage polarization-related genes. (B) ELISA analysis of cytokines, including TNF-α and IL-10. (C) Representative immunofluorescence staining images showing M1 polarization-related marker iNOS in macrophages. (D) Quantitative immunofluorescence staining-based analysis of iNOS. (E, F) Downregulated M1 polarization-related marker CD86 was detected via flow cytometry. (Adapted from [77])
